# A bibliometric and visual analysis of the impact of senescence on tumor immunotherapy

**DOI:** 10.3389/fimmu.2025.1566227

**Published:** 2025-04-11

**Authors:** Zixu Liu, Yuchen Mao, Shukai Wang, Haoyu Zheng, Kangping Yang, Liang Yang, Peng Huang

**Affiliations:** ^1^ Center for Evidence-Based Medicine, School of Public Health, Jiangxi Medical College. Nanchang University, Nanchang, China; ^2^ Jiangxi Province Key Laboratory of Preventive Medicine, Jiangxi Medical College, Nanchang University, Nanchang, China; ^3^ First Clinical Medicine School, Nanchang University, Nanchang, China; ^4^ School of Stomatology, Jiangxi Medical College, Nanchang University, Nanchang, China; ^5^ Department of Gastroenterological Surgery, The Second Affiliated Hospital, Jiangxi Medical College, Nanchang University, Nanchang, Jiangxi, China

**Keywords:** bibliometrics, visualization, senescence, aging, immunotherapy, cancer

## Abstract

**Background:**

Recently, many studies have focused on the relationship between senescence and immunotherapy in cancer treatment. However, relatively few studies have examined the intrinsic links between the three. Whether these studies can act synergistically in the fight against cancer and the specific links between them are still unclear.

**Methods:**

We extracted, quantified, and visualized data from the literature (n = 2396) for the period 2004-2023 after rigorous quality control using citespace, GraphPad Prism, the R software package, and VOSviewer.

**Results:**

Linear fit analyses were generated to predict the number of annual publications and citations as a function of the top-performing authors, journals, countries, and affiliations academically over the past two decades such as Weiwei, Aging-us, China, and the UT MD Anderson Cancer Center. Vosviewer-based hierarchical clustering further categorized study characteristics into six clusters, including two major clusters of immunotherapy research, immunosenescence-related research factors, and timeline distributions suggesting that cellular senescence and tumor progression is a relatively new research cluster that warrants further exploration and development. Study characterization bursts and linear regression analyses further confirmed these findings and revealed other important results, such as aging (a = 1.964, R² = 0.6803) and immunotherapy (a = 16.38, R² = 0.8812). Furthermore, gene frequency analysis in this study revealed the most abundant gene, *APOE*, and SIRT1-7 proteins.

**Conclusion:**

The combination of aging therapies with tumor immunotherapies is currently in its preliminary stages. Although senescence has the greatest impact on ICB therapies, mechanistic investigations, and drug development for APOE and sirt1-7 (Sirtuins family) targets may be the key to combining senescence therapies with immunotherapies in the treatment of tumors.

## 1 Introduction

Immunotherapy has ushered in a new era of cancer treatment as a completely new paradigm. It has attracted widespread attention and spurred intensive clinical trials and basic research development by enhancing patients’ tumor immune response and precise targeting (1). Compared with conventional treatments, some patients have achieved long-term remission without cancer symptoms for years after receiving immunotherapy. For example, in a phase III trial, ipilimumab combination therapy induced durable tumor regression in patients with metastatic melanoma, and some patients even achieved complete remission (2). However, another study found that only about 27% of patients showed a significant treatment effect and the incidence of serious immune-related adverse events was as high as 10% to 15% (3). These issues suggest the need for a broader perspective to deeply understand and optimize cancer immunotherapy to overcome existing limitations and improve efficacy.

Recent studies have proposed that cellular senescence (refer to an irreversible cell cycle arrest associated with changes in cell morphology, secretory profile, and epigenetic alterations among others)plays a key role in reducing the efficacy of immunotherapies. Studies using mouse models by Damien Maggiorani et al. have demonstrated that the accumulation of senescent cells hinders cancer immunotherapy. Conversely, targeted removal of these cells through genetic or pharmacological approaches can reverse this phenotype (4). Mechanistically, senescent cell secretions promote infiltration of immunosuppressive cells, particularly myeloid-derived suppressor cells (MDSCs). These MDSCs (myeloid-derived suppressor cells) secrete inhibitory cytokines, such as TGF-β (transforming growth factor-β) and IL-10 (interleukin 10), and alter the function of antigen-presenting cells, directly or indirectly suppressing the activity of CD^8+^ T cells (Cytotoxic T Lymphocytes, CTLs), the main effector in the antitumor immune response (5). Notably, relevant studies have found that senescent cells are prevalent in cancer survivors undergoing chemotherapy and radiotherapy, further emphasizing their potential impact (6).

Cellular senescence in cell biology refers to a programmed change in cell state associated with permanent growth inhibition (7). It is usually triggered by the DNA damage response, which activates various signaling pathways such as p38 MAPK (mitogen-activated protein kinase), NF-κB (nuclear factor kappa-B), and mTOR (mammalian target of rapamycin), which induce up-regulation of NKG2D (natural killer cell group 2D) ligands and promote the recognition and clearance of senescent cells by NK and certain T cells (8). Wen Xue et al. demonstrated that reactivation of the p53 pathway efficiently induced tumor regression in a mouse model of hepatocellular carcinoma through activation of both the senescence and immune responses, revealing for the first time that cellular senescence can serve as a mechanism for permanent growth inhibition (9). With the study of the secretion of many factors, another feature of cellular senescence (10), there is increasing evidence that senescence-associated secretory phenotype (SASP) promotes T cell recruitment to clear tumors and contributes to the senescence of tumor cells, thereby limiting cancer progression. In addition, SASP promotes macrophage polarization to a tumor-suppressive M1 state, targets senescent cells, and may further induce tumor cell senescence.

This response may reflect the difference between acute and chronic senescence phenotypes, where acute senescence is usually anti-tumorigenic and participates in immune activation and clearance of senescent cells through secretion of SASPs, whereas chronic senescence may lead to a prolonged inflammatory state that attracts immune suppression cells, thus supporting tumor growth with immune escape.

However, paradoxically, cellular senescence can also protect proliferating tumor cells by affecting the tumor microenvironment (TME) through various mechanisms. Cytokines and growth factors (such as IL-6 (Interleukin-6), IL-8 (Interleukin-8), and VEGF (vascular endothelial growth factor)) contained in SASPs not only promote tumor angiogenesis but also attract immune-suppressor cells, such as myeloid-derived suppressor cells (MDSCs) and regulatory T cells (Tregs), which protect tumor cells from the immune system. In addition, the accumulation of senescent cells may prevent the immune system from removing them, leading to immune evasion (11). Therefore, cellular senescence has been considered one of the hallmarks of tumors (12). This response may reflect the difference between acute and chronic senescence phenotypes, where acute senescence is usually anti-tumorigenic and participates in immune activation and clearance of senescent cells through secretion of SASPs, whereas chronic senescence may lead to a prolonged inflammatory state that attracts immune suppression cells, thus supporting tumor growth with immune escape (13).

Thus, senescent cells, in terms of changes in surface molecules and secretagogues, can serve as potential targets for tumor immunotherapy. Senolytics and enomorphs are today’s most promising therapeutic strategies for senescence and the main ones to be combined with tumor immunotherapy. They aim to selectively remove deleterious senescent cells, modulate or inhibit the expression of chronic SASPs, enhance senescence-associated acute immune stimulatory properties, and avoid tumor-associated inflammation (14). Based on changes in senescent cell surface protein expression, such as up-regulation of PD-L1 (programmed cell death ligand 1) and down-regulation of NKG2D ligands, FDA-approved PD-1 (programmed death-1)/PD-L1 immune checkpoint blockades (ICBs) can be applied (15), or antibodies can be developed to block NKG2D ligand shedding. In addition, the expression of specific surface proteins provides methods for designing antibodies and chimeric antigen receptors (CARs) that recognize and eliminate senescent cells. For example, CAR-T (Chimeric Antigen Receptor T-Cell Immunotherapy) cells designed according to the urokinase-type plasminogen activator receptor (uPAR) by Amor et al. efficiently cleared senescent cells from post-fibrotic hepatocytes and lung tumor cells after treatment-induced senescence (TIS) (16). This also suggests that the identification of chronic senescence-specific therapeutic targets that are not present in acute senescent cells is essential. In addition, MDSCs infiltrated after senescence induction can be blocked by designing antibodies that neutralize immunosuppressive SASP factors, such as CSF-1R (Colony Stimulating Factor 1 Receptor), CCR2 (Chemokine (C-C motif) Receptor 2), and CXCR2 (C-X-C Motif Chemokine Receptor 2) inhibitors (17).

In this paper, we provide a detailed review of the relationship between senescence, and tumor immunotherapy through bibliometric analysis, and summarize the current status and hotspots of research in this field. Studies have shown that the properties of cellular senescence make it an important research direction for immunotherapy in cancer treatment. Future studies should aim at a deeper understanding of the role of senescent cells in the immune microenvironment to provide a basis for the development of new strategies for the combination of senescence therapy and tumor immunotherapy. In addition, the identification and validation of senescent cell-specific targets, as well as the development of targeted drugs, will be key directions to advance the field. We hope that this study will provide a reliable basis for knowledge expansion and innovation in related fields.

## 2 Methodology

### 2.1 Rigorous quality control and data sources

There are several mainstream databases for retrieving and storing medical information in the biomedical field, such as Web of Science (WOS), PubMed, and Scopus, and after investigating the materials and reading the literature, we finally decided to use the WOS core database as the data pool for further analysis.

There are many different types of literature in the database, and only peer-reviewed reviews and original studies with complete documentation were selected to ensure the rigor and accuracy of the articles.

### 2.2 Search strategy

After obtaining relevant title keywords and supplementing them with grid subject headings from PubMed, we conducted an exhaustive bibliographic search online via WOS via the following search format: (ALL=(“aging” OR “senescence” OR “elderly” OR “geriatrics”) AND ALL=(“cancer” OR “neoplasm” OR “carcinoma”) AND ALL=(“immunotherapy” OR “immune therapy” OR “immune checkpoint inhibitors” OR “adoptive cell therapy”).

The topic of this study was “Aging, Cancer and Immunotherapy”; the search period was from January 1, 2004, to December 31, 2023; the article type was selected as review and article; and the language of the article was selected as English.

### 2.3 Data collection

The filtering function of the database was utilized to restrict the period of the study and the type of articles and to exclude non-English literature to ensure the stability of the subsequent analysis. A large amount of data related to lymphoma metabolism, including year of publication, references, authors, country, affiliation, journals, global citations, local citations, total publications, total citations, average citations, non-self-citing citations, the h-index, the g-index, and keywords, were mined from the above literature, quantified and deposited into Microsoft Excel. During the data collection process, one author was designated the primary data collector, and another author was responsible for further checking the data to avoid errors and bias in the data collection process.

### 2.4 Interpretation of indicators

Publications and citations are standard metrics used in scientometrics to measure scholarly output, with publications indicating scholarly output and citations indicating scholarly impact and contribution. Global citations of an article indicate how often the article is cited in the entire WOS database. Local citations of an article indicate how often the article is cited in the current dataset domain.

The h-index, m-index, and g-index are three hybrid metrics that can be synthesized to evaluate academic performance. The h index is a measure of a scholar’s productivity and impact. Specifically, the h-index of a scholar means that he/she has h papers, each of which has been cited at least h times. The h index can consider both the number of papers and the number of citations, which reflects the influence of a scholar in a more comprehensive way. It does not consider papers with more than h citations, nor does it consider papers with fewer than h citations, which may not be accurate enough to evaluate scholars in some fields. To address the limitations of the h index, we introduce the g index, which takes into account the sum of the squares of the number of citations of a paper. The g index can better reflect the influence of highly cited papers, avoiding the single criterion of the h index, which focuses only on the h citations. In addition, to make a fairer comparison between the impact of emerging and established scholars, we introduce the m-index, which is a time-weighted version of the h-index that takes into account the growth of a researcher’s impact throughout his or her career. It is the h-index divided by the number of years of experience of the researcher, which more accurately reflects the impact of younger or quality-oriented scholars.

### 2.5 Scientific bibliometrics and visual analytics

Further visualization and analysis were achieved with VOSviewer 1.6.20(0), GraphPad Prism 9, R 4.2.2, and the online graphing software Chiplot. The R language provides powerful statistical and data analysis capabilities, a wealth of visualization options, and extensive community support, with the R packages (“bibliometrix”, “ggplot2”, “circlize”, and “tmap”) playing important roles. bibliometrix”, ‘ggplot2’, ‘circlize’, and ‘tmap’) play important roles. Important role. Visual networks are constructed and generated through coauthorship and cooccurrence analysis via VOSviewer, a program based on the Java environment (18, 19). Node values in a visual network indicate total link strength (TLS), the thickness of a line indicates the strength of the relationship between two individuals, and the color of a node indicates a different clustering or period. The chip lot provides several features for the visual analysis of data, clustering analysis, and prediction accordingly for countries, institutions, journals, author keywords, and other institutions.

### 2.6 Linear regression, nonlinear regression and statistical analysis

Linear regression analysis is a statistical method used to study the linear relationship between an independent variable and a dependent variable to facilitate quantitative analysis. Its core purpose is to develop a regression model to facilitate the prediction and explanation of changes in the dependent variable. In this work, using time as the independent variable and the number of article citations as the dependent variable, linear regression analysis is used to determine whether there is a dependency between the two variables, generate regression curves to predict future trends, calculate the goodness of fit, and determine whether there is statistical significance between the two variables. p values reflect the magnitude of the likelihood of the event occurring, p < 0.05 indicates a statistically significant difference, p < 0.01 indicates a statistically significant difference, and P < 0.001 indicates a statistically extremely significant difference. Nonlinear regression analysis is a statistical method used to address nonlinear relationships between dependent and independent variables. Afterward, the logit model of the Sigma–Aldrich 4XL model was used to analyze the corresponding data via nonlinear regression analysis, and the obtained data were subjected to model evaluation and model diagnosis, which all met the corresponding requirements.

In this study, the corresponding data collected by VoSviewer for different years were made into Excel tables, and the corresponding linear and nonlinear analyses were performed via Prism 9.

**Figure 1:**
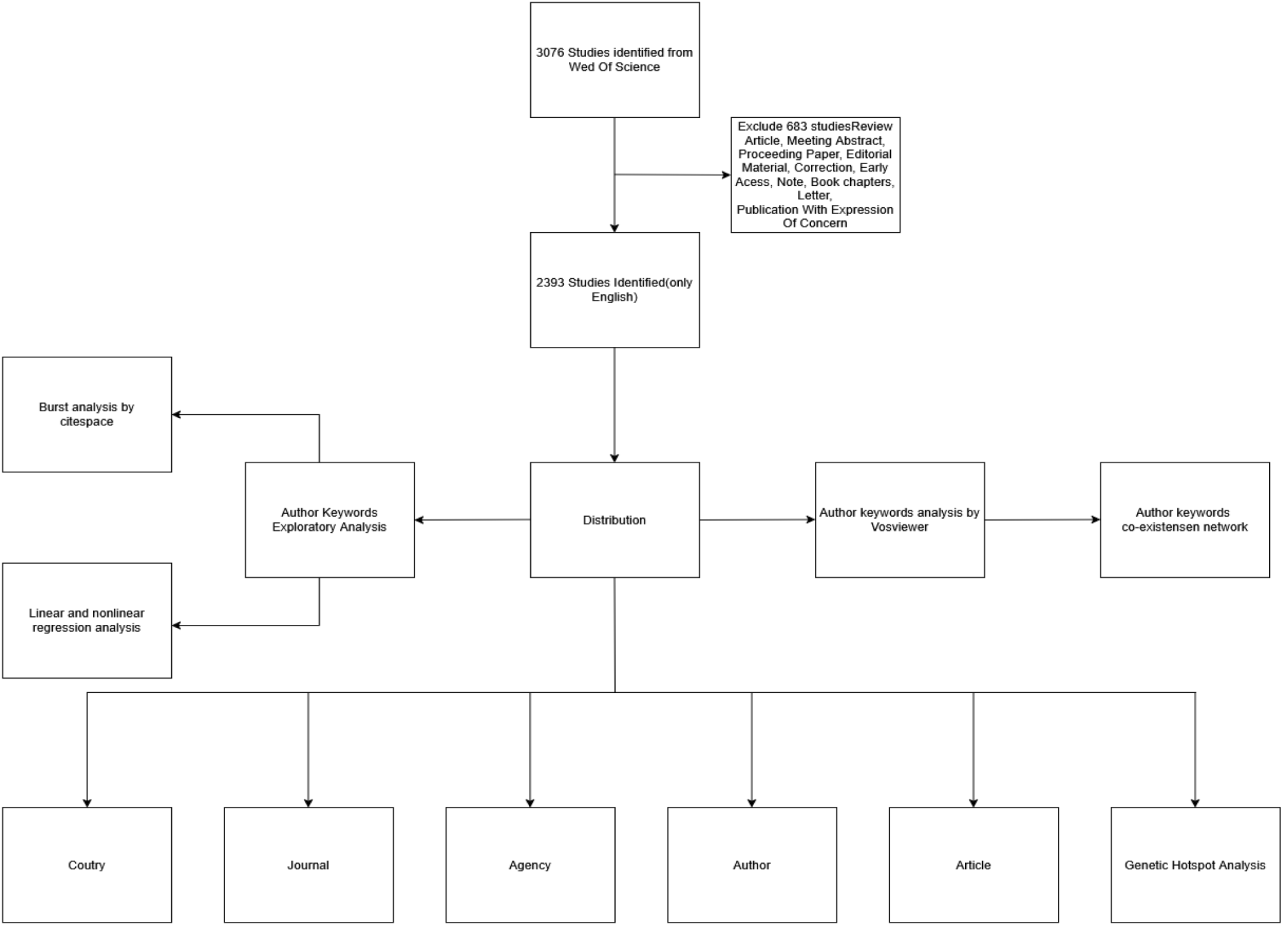
Bibliometrics Overall Flowchart.

The above is shown in **Figure 1**: Bibliometrics Overall Flowchart.

## 3 Results

### 3.1 Overview of cancer, aging, and immunotherapy research areas

The statistical summary of our database shows in **Table 1** that in terms of published literature in this field, the average year of publication is 4.57 years, and the number of publications continues to grow at a rate of 1.8% per year. At the level of international co-authorship, the international co-authorship rate in this field is 23.02%.

**Table 1:** Basic characteristics of the data pool.

Description	Results
Timespan	2004 to 2023
Journals	690
Documents	2576
article	1786
review	631
References	115548
Authors	20455
Keywords plus	5342
Author’s keywords	4622
Annual growth rate	1.8%
Document average age	4.57
Average citations per document	34.59
Authors of single-authored documents	46
Coauthors per document	11.9
International Co-Authorship	23.02%

**Figure 2:**
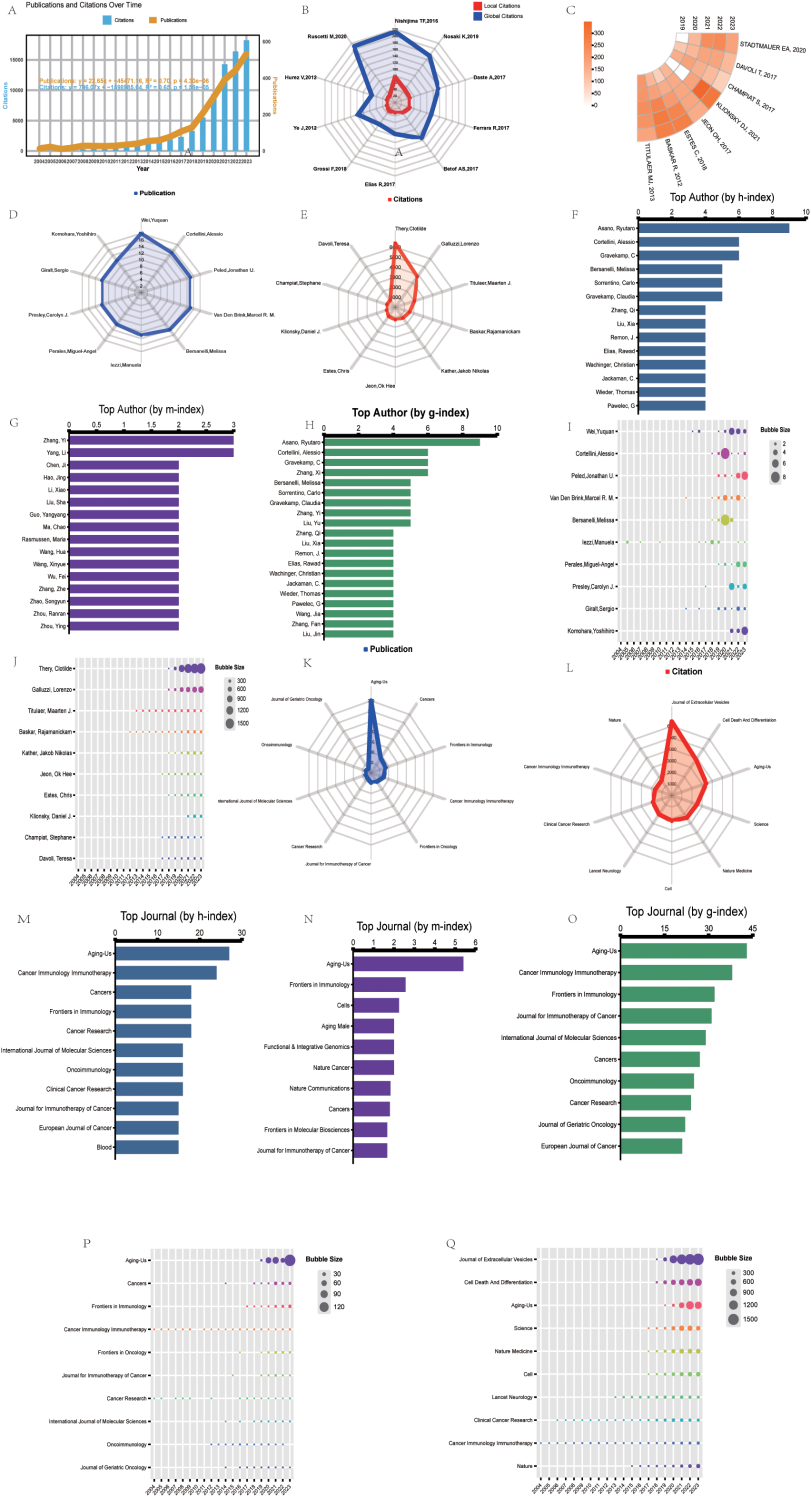
Top 10 most cited papers, productive authors, and journals in bibliometrics and visualization research. **(A)** Timeline and predictor function for the number of publications (orange) and citations (blue) for studies on aging and cancer immunotherapy. R^2^ indicates the correlation coefficient. P-values represent statistical differences. **(B)** Radar plots showing local (red) and global (blue) citation rankings and the corresponding number of the top ten most cited papers. Global citations indicate the number of times the paper was cited by all documents in the entire WOS database. Local citations indicate the number of times the paper was cited in the WOS core database. **(C)** The ring heat map visualizes the number of citations per year for each of the top-cited papers over the last five years. **(D)** Publication rankings and the corresponding number of top ten authors. **(E)** Ranking of citations and corresponding number of top ten authors. **(F)** h-index ranking and a corresponding number of top ten authors. **(G)** m-index ranking and the corresponding number of top ten authors. **(H)** g-index ranking and the corresponding number of top ten authors. **(I)** Annual number of papers published by the top ten authors. **(J)** The annual number of citations for the top ten authors. **(K)** Publication rank and the corresponding number of top ten most productive journals. **(L)** Citation rank and a corresponding number of the top ten most productive journals. **(M)** h-index ranking and the corresponding number of the top ten most productive journals. **(N)** m-index ranking and a corresponding number of top ten journals. **(O)** g index ranking and the corresponding number of top ten journals. **(P)** Number of annual publications of the top ten journals in terms of publications. **(Q)** Annual number of citations for journals ranked in the top ten journals for citations.

**Figure 2A** summarizes the number of publications and citations per year in the fields of cancer, aging, and immunotherapy research from 2004–2023. The annual number of publications or citations in the field generally showed a steady upward trend from 2004–2023. Two prediction functions were further developed via linear regression analysis to predict future trends in publications and citations. The function used to predict the number of publications per year was “: y = 22.65x–45471.16”, with a correlation coefficient of R² = 0.70. In addition, the function used to predict the number of citations per year was “y = 796.07x -1598985.04”, with a correlation coefficient of R² = 0.65. The steady increase in the number of publications and citations suggests that the fields of cancer, aging, and immunotherapy research have received increased academic attention and flourished over the past two decades.

### 3.2 Core content of landmark literature based on the highest number of local citation

In the literature, citation analysis, global citations reflect a paper’s total number of citations in the entire WOS database, whereas local citations represent its citation frequency in the core WOS database. In contrast, local citations are a more accurate indicator of a paper’s influence among core scholars in a particular research area, and papers with high local citation rates are usually regarded as key literature in the field. Among the top ten papers in terms of local citations (**Figure 2B**), the article authored by Nishijima TF ranked first, with 79 citations, showing its significant recognition and influence in the research field. It is closely followed by the paper by Nosaki K, which has been cited 49 times. These figures not only reveal how highly regarded both authors are in their respective fields but also reflect the value and widespread impact of their work. Notably, the annual citations of the top ten most locally cited papers have increased significantly over the past year (**Figure 2C**). This trend, which is more prominent than it was in the previous period, indicates the growing academic attention and interest in the relevant research area and signals the potential for future development of the field and the potential importance of scholarly contributions. According to the top 25 references with the strongest citation bursts (**Table 2**), these findings were published in high-impact journals in the field such as The New England Journal of Medicine (40%), The Lancet (12%), Science (8%), Cancer Treatment Reviews (8%), Journal of Experimental & Clinical Cancer Research (4%), Cell Reports (4%), CA: A Cancer Journal for Clinicians (4%), Nature Medicine (4%), Physiological Reviews (4%), Nature (4%), Cancer Discovery (4%), and Cell (4%). An in-depth analysis revealed that the vast majority of the literature (84%) focused directly on using immune checkpoint therapies in cancer. Of these 21 publications, 15 examined the efficacy of immune checkpoint inhibitors and their clinical studies, mainly exploring the effectiveness of different inhibitors (mainly PD-1 and PD-L1 blockers) in a variety of cancers, such as non-small-cell lung cancer, melanoma, etc., and comparing the efficacy and safety of different PD-1 monoclonal antibodies, as well as the effect of combination therapy. The remaining six analyzed factors affecting the effectiveness of immune checkpoint blockade therapies, such as tumor genomic mutations, immune senescence, and signaling in the tumor microenvironment. These studies screened applicable patients with predictive models and biomarkers to optimize treatment efficacy. In addition, four other papers provide new perspectives and therapeutic targets on cancer and cellular senescence, offering new ideas for individualized treatment and the combination of senescence therapy and immunotherapy. These results indicate that optimizing the application of immune checkpoint therapies in cancer treatment is a core research topic in the field at present, and provides important guidance and reference for future basic research and clinical practice through multilevel exploration.

**Table 2:** Top 25 references with strongest citation bursts.

References	Title	Year	Strength	Begin	End	IF
Topalian SL, 2012, NEW ENGL J MED, V366, P2443, DOI 10.1056/NEJMoa1200690, DOI	Safety, activity, and immune correlates of anti-PD-1 antibody in cancer	2012	13.09	**2014**	2017	96.2
Borghaei H, 2015, NEW ENGL J MED, V373, P1627, DOI 10.1056/NEJMoa1507643, DOI	Nivolumab versus Docetaxel in Advanced Nonsquamous Non-Small-Cell Lung Cancer	2015	28.6	**2016**	2020	96.2
Brahmer J, 2015, NEW ENGL J MED, V373, P123, DOI 10.1056/NEJMoa1504627, DOI	Nivolumab versus Docetaxel in Advanced Squamous-Cell Non-Small-Cell Lung Cancer	2015	21.19	**2016**	2020	96.2
Larkin J, 2015, NEW ENGL J MED, V373, P23, DOI 10.1056/NEJMoa1504030, DOI	Combined Nivolumab and Ipilimumab or Monotherapy in Untreated Melanoma	2015	15.59	**2016**	2020	96.2
Robert C, 2015, NEW ENGL J MED, V372, P320, DOI 10.1056/NEJMoa1412082, DOI	Nivolumab in previously untreated melanoma without BRAF mutation	2015	15.24	**2016**	2020	96.2
Robert C, 2015, NEW ENGL J MED, V372, P2521, DOI 10.1056/NEJMoa1503093, DOI (20)	Pembrolizumab versus Ipilimumab in Advanced Melanoma	2015	12.89	**2016**	2019	96.2
Rizvi NA, 2015, SCIENCE, V348, P124, DOI 10.1126/science.aaa1348, DOI	Mutational landscape determines sensitivity to PD-1 blockade in non-small cell lung cancer	2015	12.45	**2016**	2020	44.7
Motzer RJ, 2015, NEW ENGL J MED, V373, P1803, DOI 10.1056/NEJMoa1510665, DOI (21)	Nivolumab versus Everolimus in Advanced Renal-Cell Carcinoma	2015	11.06	**2016**	2020	96.2
Postow MA, 2015, NEW ENGL J MED, V372, P2006, DOI 10.1056/NEJMoa1414428, DOI	Nivolumab and ipilimumab versus ipilimumab in untreated melanoma	2015	9.96	**2016**	2019	96.2
Garon EB, 2015, NEW ENGL J MED, V372, P2018, DOI 10.1056/NEJMoa1501824, DOI	Pembrolizumab for the treatment of non-small-cell lung cancer	2015	9.67	**2016**	2020	96.2
Reck M, 2016, NEW ENGL J MED, V375, P1823, DOI 10.1056/NEJMoa1606774, DOI	Pembrolizumab versus Chemotherapy for PD-L1-Positive Non-Small-Cell Lung Cancer	2016	20.95	**2017**	2021	96.2
Herbst RS, 2016, LANCET, V387, P1540, DOI 10.1016/S0140-6736(15)01281-7, DOI	Pembrolizumab versus docetaxel for previously treated, PD-L1-positive, advanced non-small-cell lung cancer (KEYNOTE-010): a randomized controlled trial	2016	13.97	**2017**	2020	98.4
Nishijima TF, 2016, CANCER TREAT REV, V45, P30, DOI 10.1016/j.ctrv.2016.02.006, DOI	Comparison of efficacy of immune checkpoint inhibitors (ICIs) between younger and older patients: A systematic review and meta-analysis	2016	13.36	**2017**	2021	9.6
Rittmeyer A, 2017, LANCET, V389, P255, DOI 10.1016/S0140-6736(16)32517-X, DOI	Atezolizumab versus docetaxel in patients with previously treated non-small-cell lung cancer (OAK): a phase 3, open-label, multicenter randomized controlled trial	2017	13.27	**2017**	2020	98.4
Fehrenbacher L, 2016, LANCET, V387, P1837, DOI 10.1016/S0140-6736(16)00587-0, DOI	Atezolizumab versus docetaxel for patients with previously treated non-small-cell lung cancer (POPLAR): a multicenter, open-label, phase 2 randomized controlled trial	2016	9.31	**2017**	2020	98.4
Sileni VC, 2014, J EXP CLIN CANC RES, V33, P0, DOI 10.1186/1756-9966-33-30, DOI	Efficacy and safety of ipilimumab in elderly patients with pretreated advanced melanoma treated at Italian centers through the expanded access programme	2014	8.4	**2017**	2019	11.4
Ferrara R, 2017, CANCER TREAT REV, V60, P60, DOI 10.1016/j.ctrv.2017.08.003, DOI	Immunosenescence and immunecheckpoint inhibitors in non-small cell lung cancer patients: Does age really matter?	2017	8.38	**2018**	2021	9.6
Sharma P, 2015, SCIENCE, V348, P56, DOI 10.1126/science.aaa8172, DOI	The future of immune checkpoint therapy	2015	8.34	**2018**	2020	44.7
Charoentong P, 2017, CELL REP, V18, P248, DOI 10.1016/j.celrep.2016.12.019, DOI	Pan-cancer Immunogenomic Analyses Reveal Genotype-Immunophenotype Relationships and Predictors of Response to Checkpoint Blockade	2017	9.78	**2021**	2022	7.5
Sung H, 2021, CA-CANCER J CLIN, V71, P209, DOI 10.3322/caac.21660, DOI	Global cancer statistics 2020: GLOBOCAN estimates of incidence and mortality worldwide for 36 cancers in 185 countries	2021	33.22	**2022**	2024	503.1
Jiang P, 2018, NAT MED, V24, P1550, DOI 10.1038/s41591-018-0136-1, DOI	Signatures of T cell dysfunction and exclusion predict cancer immunotherapy response	2018	18.64	**2022**	2024	58.7
Calcinotto A, 2019, PHYSIOL REV, V99, P1047, DOI 10.1152/physrev.00020.2018, DOI	Cellular Senescence: Aging, Cancer, and Injury	2019	12.59	**2022**	2024	29.9
Mariathasan S, 2018, NATURE, V554, P544, DOI 10.1038/nature25501, DOI	TGFβ attenuates tumour response to PD-L1 blockade by contributing to exclusion of T cells	2018	11.81	**2022**	2024	50.5
Hanahan D, 2022, CANCER DISCOV, V12, P31, DOI 10.1158/2159-8290.CD-21-1059, DOI (12)	Hallmarks of Cancer: New Dimensions	2022	10.37	**2022**	2024	29.7
Gorgoulis V, 2019, CELL, V179, P813, DOI 10.1016/j.cell.2019.10.005, DOI	Cellular Senescence: Defining a Path Forward	2019	8.53	**2022**	2024	45.5

### 3.3 Scientometric and visualization studies of the top ten most prolific authors

Identifying key authors and their influence in a given research field by analyzing the contributions and collaborative networks of distinguished scholars in a multifaceted way is important for tracking cutting-edge developments in the field. After a comprehensive analysis, the results show that two scholars, Cortellini, Alessio, and Casasnovas Olivier, consistently rank among the top five in three indicators: literature output, the h-index, and the g-index (**Figures 2D-H**). Trend analysis of annual publication volume revealed that the publication volume of the vast majority of the top ten authors (approximately 90%) significantly increased in the last five years, especially Cortellini, Alessio, and Casasnovas Olivier, who each experienced a significant publication peak during this period (**Figure 2I**). In addition, the yearly changes in the top ten authors in terms of citations show that Thery, Clotilde’s citations have grown rapidly in recent years, creating a clear surge trend. In contrast, Titulaer, Maarten J., and Baskar, Rajamanickam’s annual citation changes have been relatively stable, showing the persistence of their long-term impact (**Figure 2J**).

### 3.4 Scientometric and visualization studies of the ten most productive journals

Choosing a high-quality academic journal to publish your manuscript can not only expand the audience of your research but also enhance your academic impact. After a comprehensive analysis, the journal Aging-Us demonstrated excellence in several evaluation metrics, ranking first in all the metrics except for the number of cited studies, where it ranked third. In addition, Frontiers in Immunology, Cancers, and the Journal for Immunotherapy of Cancer also performed solidly in the top ten on several other important metrics, despite having a lower total volume of literature published (**Figures 2K-O**). A look at the annual publication trends of the ten most productive journals shows that Cancer Immunology Immunotherapy has been relatively stable in terms of publication volume, whereas Aging-Us, Cancers, and Frontiers in Immunology have shown significant growth over the past five years (**Figure 2P**). Analysis of annual citation changes for the top ten cited journals revealed that Cancer Immunology Immunotherapy has remained stable in terms of citations each year, whereas the Journal of Extracellular Vesicles, Cell Death and Differentiation, and Aging-Us, on the other hand, citation frequency has continued to increase over the past five years (**Figure 2Q**). These results indicate that Aging-Us, Cancers, and the Journal for Immunotherapy of Cancer have an excellent presence in the relevant academic field and are ideal for publishing high-quality research, providing a platform for wider academic dissemination of research.

### 3.5 Scientometric and visualization studies of author linkages

**Figure 3:**
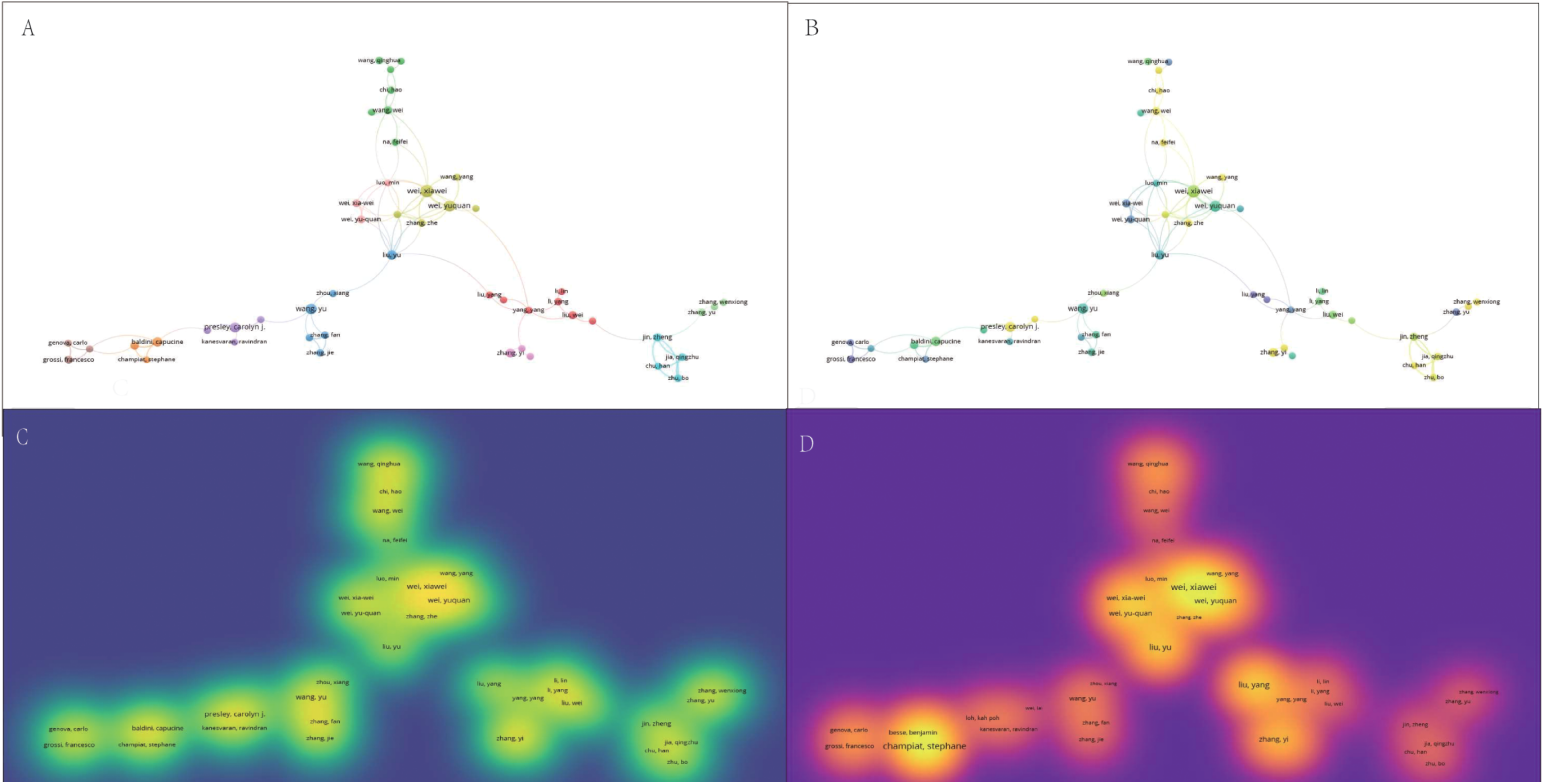
Multivariate visualization network of links between authors. **(A)** Hierarchical cluster analysis to categorize authors into seven groups based on their inter-author associations. Cluster 1: grass green; Cluster 2: purple; Cluster 3: red; Cluster 4: blue; Cluster 5: coffee; Cluster 6: green; Cluster 7: orange; Cluster 8: cyan. **(B)** Timeline distribution of author clusters. The purple nodes indicate earlier appearances, and the yellow nodes indicate later appearances. **(C)** Interauthor publication density visualization. **(D)** Interauthor citation density visualization.

Hierarchical cluster analysis reveals the complex structure and impact of collaborative networks among researchers, providing key insights for understanding patterns of academic collaboration. Studying the evolution of different clusters over time and the differences in publication and citation densities is an important guide for future research collaboration and citation strategies. Using hierarchical cluster analysis, the study divided the 20,455 authors into eight clusters based on their interconnections (**Figure 3A**): the first group was grass-green; the second group was purple; the third group was red; the fourth group was blue; the fifth group was coffee-colored; the sixth group was green; the seventh group was orange; and the eighth group was cyan. Within these clusters, Wei, Xiawei, Wang, Yu, Zhang, Yi, Presley, Carolyn J, Wei, Xiawei, Wang, We, Jin, Zheng, Wang, and Kai were identified as key nodes in the collaborative network. The timeline of the author groups revealed that the third cluster emerged earlier and gained longevity, whereas the seventh cluster emerged more recently (**Figure 3B**). Visualization of publication density among authors shows that clusters 1, 2, and 5 publish many more than the other clusters do (**Figure 3C**). In terms of citation density, clusters 1 and 4 clearly lead in terms of the number of citations (**Figure 3D**). This study reveals the collaborative structure between different author clusters and their citation performance, and the combined academic performance of Cortellini, Alessio, Casasnovas Olivier, Thery, Clotilde, and Cluster 1 can be drawn upon for subsequent studies to keep up with cutting-edge advances in the field.

### 3.6 The comprehensive performance and development evolution of the top ten most productive countries and affiliations

**Figure 4:**
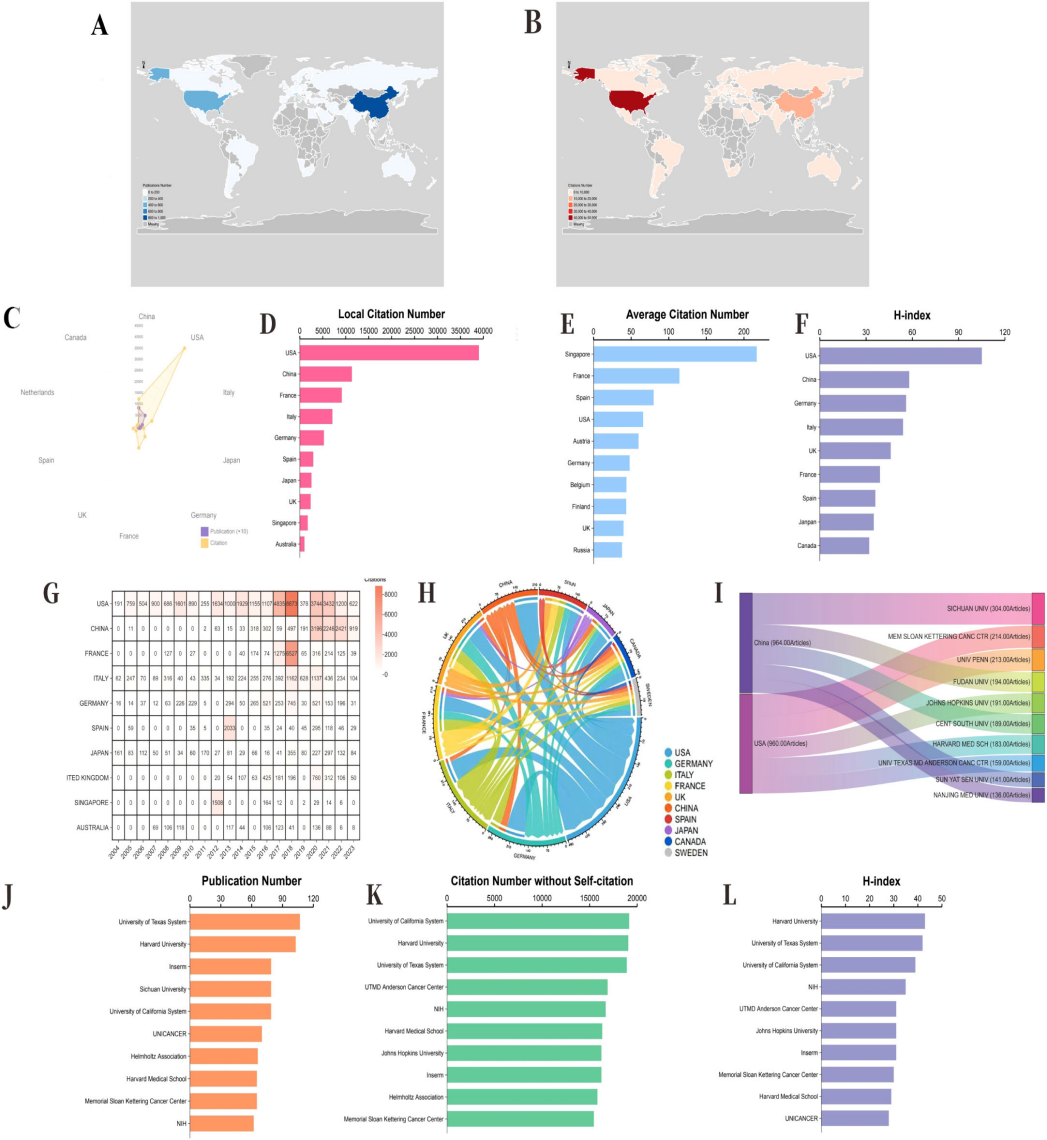
Combined performance and development evolution of the top 10 countries and affiliations in terms of productivity. **(A)** Geographical distribution of publications by country. **(B)** Geographical distribution of citation volumes in different countries. **(C)** The top ten countries with the highest production in terms of publication volume (purple) and citation volume (orange). **(D)** The number of citations for local self-citation rankings and the corresponding number for the top ten most productive countries. **(E)** The average citation ranking and the corresponding number of the top ten most productive countries. **(F)** The corresponding number of h-index rankings and the top ten countries with the highest productivity. **(G)** Cumulative citation ranking and the corresponding number of the top ten most productive countries. **(H)** Circos diagram displaying the partnership network between different countries. **(I)** On the left, the Sankey diagram displays the country sources and total citations of the top ten most productive affiliations. Right, which refers to the total publications and annual publications of the corresponding affiliated institutions. **(J)** Publication numbers and ranks of the top ten most productive affiliations. Inserm, France National Institute of Health and Medical Research (French, Institut national de la santé et de la recherche médicale) NIH, National Institutes of Health (USA) **(K)** The number of citations without self-citation and the top ten most productive affiliates. **(L)** The corresponding number of h-index levels and the top ten most productive affiliations. UTMD, University of Texas Medical Branch.

The global distribution trends of publications and citation volumes indicate a high degree of consistency in these metrics across different countries, with the United States and China consistently occupying dominant positions, ranking first and second, respectively (**Figures 4A-C**). Additionally, the United States, France, Germany, Spain, and the United Kingdom also perform prominently in other metrics, including citation counts exclusive of self-citations, average citations per article, and the h-index, consistently ranking within the top ten (**Figures 2D-F**). Cumulative citation rankings and data from the top ten most productive countries further confirm the United States’ leading position, significantly outpacing other nations. However, since 2020, China has experienced explosive growth in annual citations, surpassing the United States in 2022 (**Figure 4G**). The Circos diagram illustrates complex and close collaborative relationships among countries (**Figure 4H**). Regarding the most productive institutions, data show that Sichuan University in China, the Memorial Sloan Kettering Cancer Center, and the University of Pennsylvania in the United States occupy the top three positions. Further analysis of annual publications reveals that these three institutions experienced slow growth before 2020 but have shown significant increases since then (**Figure 4I**). Moreover, the University of Texas, Harvard University, and the University of California in the United States consistently rank in the top five across other metrics, such as publication number, citation number without self-citation, and the h-index (**Figures 4J-L**). These results suggest that the focus of global research output is shifting, with China markedly increasing its influence on the international academic community in recent years, whereas the United States continues to maintain its traditional leadership in academia. Through robust international collaboration and the continuous enhancement of research capabilities, academic exchanges among countries are expected to deepen further, fostering the vigorous development of global scientific research.

### 3.7 Country and institutional cluster analysis

**Figure 5:**
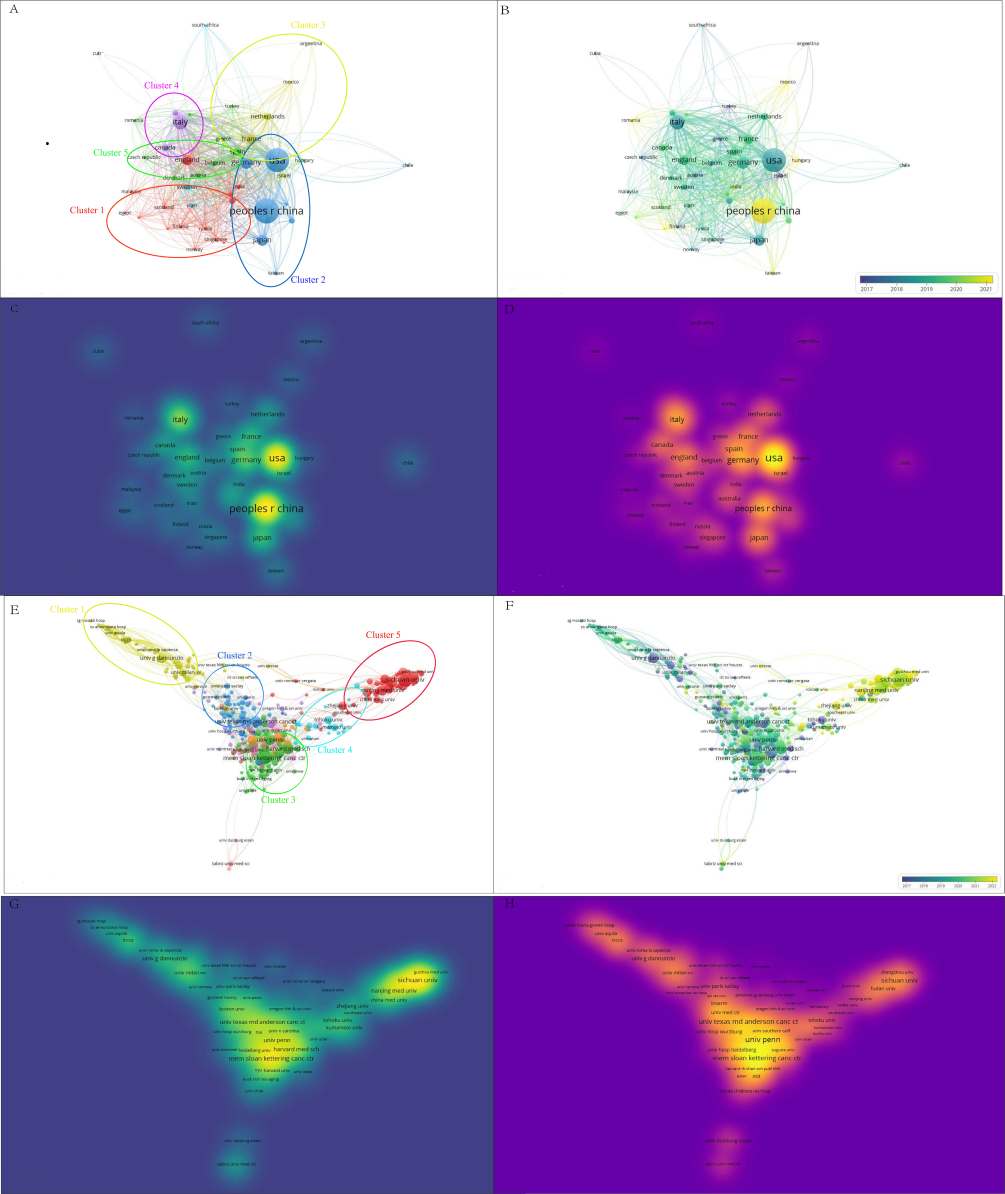
A bibliometric visualization study of countries and affiliations. **(A)** Hierarchical cluster analysis divides the countries into five clusters based on the overall strength of ties. Cluster 1: red; Cluster 2: blue; Cluster 3: yellow; Cluster 4: purple; Cluster 5: green. **(B)** Timeline distribution of the country cluster analysis, with yellow nodes indicating newer occurrences and purple nodes indicating earlier occurrences **(C)** Visualization of inter-country publication density. **(D)** Visualization of citation density between countries. **(E)** Hierarchical cluster analysis with affiliations categorized into five clusters based on overall strength of ties. Cluster 1: green; Cluster 2: blue; Cluster 3: yellow; Cluster 4: cyan; Cluster 5: red. **(F)** Timeline distribution of the association cluster analysis. Yellow nodes indicate newer occurrences and purple nodes indicate earlier occurrences, **(G)** Density of visualized publications between affiliations. **(H)** Citation density between visualized associations.

After hierarchical cluster analysis, the 44 countries were divided into clusters on the basis of intercountry linkages (**Figure 5A**). Cluster 1: red; Cluster 2: blue; Cluster 3: yellow; Cluster 4: purple; Cluster 5: green. The United States and China, the two countries with the most publications, are located in cluster 2 of these five clusters. Among the five clusters, England (TLS = 385), the United States (TLS = 680), France (TLS = 310), Italy (TLS = 366), Spain (TLS = 277), and Germany (TLS = 407) are the key nodes of the cooperative network. The timeline distribution of the country clusters shows that Cluster 2 appeared earlier and has been developing for a longer time, whereas Clusters 1 and 3 appeared later, while each cluster has countries with emerging momentum, represented by China in Cluster 2, which appeared late but developed rapidly (**Figure 5B**). Visualization of the publication density between countries reveals that cluster 2 has a greater number of publications than the other clusters do (**Figure 5C**). Visualization of the citation density between countries reveals that cluster 2 has a greater number of citations than the other clusters do, followed by cluster 3 (**Figure 5D**). After hierarchical cluster analysis, the 343 affiliations were divided into several clusters on the basis of the links between affiliations (**Figure 5E**). Cluster 1: yellow; Cluster 2: blue; Cluster 3: green; Cluster 4: cyan; Cluster 5: red. Among these five clusters, G d’Annunzio University of Chieti-Pescara (TLS=139), The University of Texas MD Anderson Cancer Center (TLS=151), Memorial Sloan Kettering Cancer Center (TLS=182), Hokkaido University (TLS=42), and Shanghai Jiao Tong University (TLS=62) are key nodes for network collaboration. The distribution of the clusters on the timeline shows that Cluster 1 appeared earlier and has been developing for a longer period of time, whereas Cluster 5 appeared later has more publications, and is developing well (**Figure 5F**). The visualization of publication density among institutions shows that the number of publications in Cluster 3 is greater than that in other clusters (**Figure 5G**), but Sichuan University is the institution with the greatest number of publications in Cluster 3. The visualization of citation density among institutions reveals that the number of citations in Cluster 3 is greater than that in other clusters (**Figure 5H**). Cluster 4 has more comprehensive academic performance, which can be used as a reference for subsequent studies to grasp the cutting-edge progress in this field.

### 3.8 Author keyword cluster analysis

**Figure 6:**
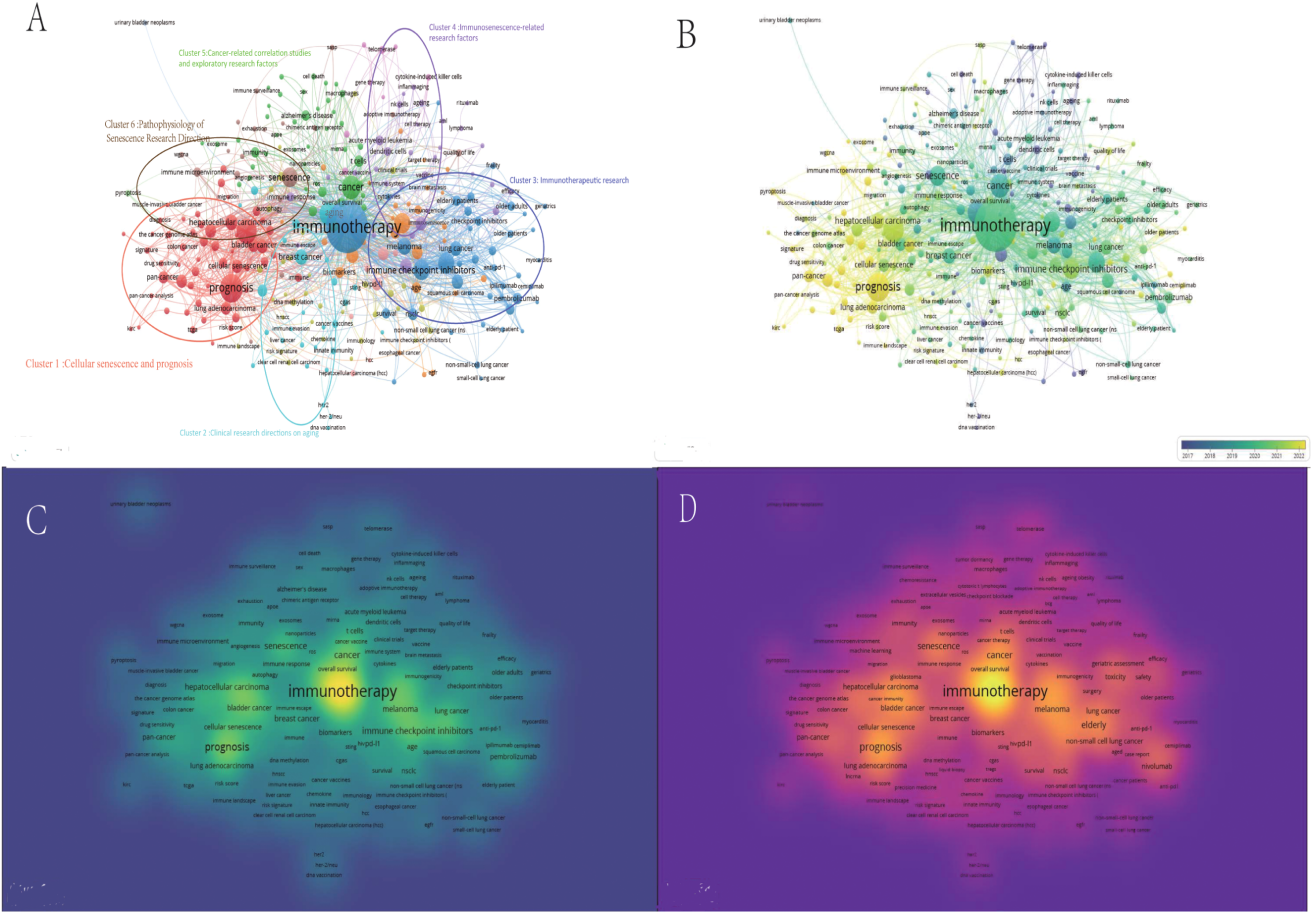
A multinetwork visualization study of research hotspots related to aging, cancer, and immunotherapy. **(A)** Visual clustering study of author keywords on the basis of co-occurrence frequency. Node values indicate co-occurrence frequency. Cluster 1 (red): Cluster 1: Cellular senescence and prognosis. Cluster 2 (cyan): Cluster 2: Three cancers related to aging in the research area: breast, colorectal, and liver cancers. Cluster 3 (blue): Cluster 3: Immunotherapeutic research(TLS = 3915, TO = 1475).Cluster 4 (purple): Cluster 4: Immunosenescence-related research factors Cluster 5 (purple): Cluster 5: Cancer-related correlation studies and exploratory research factors Cluster 6 (brown): Cluster 6: Pathophysiology of Senescence Research Direction **(B)**: Timeline distribution of author keywords. The purple nodes indicate earlier occurrences, and the yellow nodes indicate later occurrences. **(C)** Visualization of keyword connection density with other keywords. **(D)** Visualization of keyword occurrence density. A hierarchical clustering strategy was used to visualize the clustering study of author keywords on the basis of co-occurrence frequency **(A)**. All author keywords appeared at least 5 times, and 293 items were finally generated and used for the clustering study. These items were categorized into 12 clusters, with the first six clusters further defined and marked with circles (Cluster 1 (red): Cluster 1: Prognosis and its associated risk factors, with a particular focus on the tumor microenvironment. Cluster 2 (cyan): Cluster 2: Three cancers related to aging in the research area: breast, colorectal, and liver cancers. Cluster 3 (blue): Cluster 3: A group of drugs, cancer types, and patients related to the TME. Cancer types, and patients related to immunotherapyacy. Cluster 4 (purple): Cluster 4: Immunosenescence, its potential causes and the factors that may affect Cluster 5 (purple): Cluster 5: Cancer-related correlation studies and exploratory research factors; Cluster 6 (brown): Cluster 6. Pathophysiology of Senescence Research Direction).

After some confounding terms were excluded, senescence (TLS = 247), immune checkpoint inhibitors (TLS = 238), biomarkers (TLS = 204), immunosenescence (TLS = 151), cellular senescence (TLS = 106), and t-cells (TLS = 76) were identified as key nodes in the collaborative network between these six clusters. In addition, the keywords prognosis (TLS = 482) and tumor microenvironment (TLS = 373) were identified as the two keywords with the highest total link strength among all the keywords after some confounders were excluded as shown in **Figure 6**.

The results of the research hotspot multinetwork visualization revealed that research is currently focused on immunotherapeutic research (cluster 3), mainly immunotherapy research on drugs, receptors and diseases, which include pd-1 (TLS = 233), immune checkpoint inhibitors (TLS=238), nivolumab (TLS=90), anti-pd-1 (TLS=61), pembrolizumab (TLS=89), ipilimumab (TLS=49), cemiplimab (TLS=18), and others. TLS=18) and other drug target receptors as well as the corresponding drugs.

The diseases were mainly non-small cell lung cancer (TLS=260) and melanoma (TLS=178), and the irrelevant option of myocarditis (TLS=8) was excluded. The above results revealed the main drug targets and major cancer types in the fields of cancer and aging immunotherapy, which are worthy of further investigation in clinical research and basic research.

The temporal distribution of keyword clusters revealed that cellular senescence and prognosis (cluster 1) is a relatively new research cluster, and the main links with cellular senescence are the tumor microenvironment (TLS=373), biomarkers (TLS=87), immune infiltration (TLS=9), and immunotherapy (TLS=10).), immune infiltration (TLS=98), and machine learning (TLS=45) methods.

The main links to prognosis within the cluster and more recent advances are machine learning (TLS=45), immune infiltration (TLS=98), the tumor microenvironment (TLS=373), and pan-cancer (TLS=87). The most significant linkage relationship outside the cluster was the corresponding relationship with cancer and immunotherapy (link strength=68).

Cluster 6, Pathophysiology of Senescence Research Direction, is likewise one of the hotspots of past research. Key node senescence (TLS=247) is closely related to core immunotherapy (TLS=1617) (link strength=32), and other keywords include the immune microenvironment (TLS=46), quality of life (TLS =42), and the microenvironment (TLS=25).

### 3.9 An explosive analysis of the characteristics of studies on the relationships between senescence or aging, cancer, and immunotherapy

The Explosive analysis indicated that “cancer immunotherapy” was the earliest keyword with the highest burst intensity and the longest duration, whereas “non-small cell lung cancer” was the most explosive. The most frequently occurring keywords were “checkpoint,” “blockade,” “immune-related adverse events,” and “prognostic model.” The term “prognostic model” has recently emerged as a significant area of interest. To the best of our knowledge, the latest research directions in this field are related to the tumor immune microenvironment and senescence (1–3). As the keyword cluster analyzed by CiteSpace is not strictly author keywords and there are many interfering terms, the four keywords “cancer immunotherapy,” “checkpoint blockade,” “immune-related adverse events,” and “prognostic model” were selected as the most relevant terms. The frequency was analyzed both linearly and nonlinearly.

**Figure 7:**
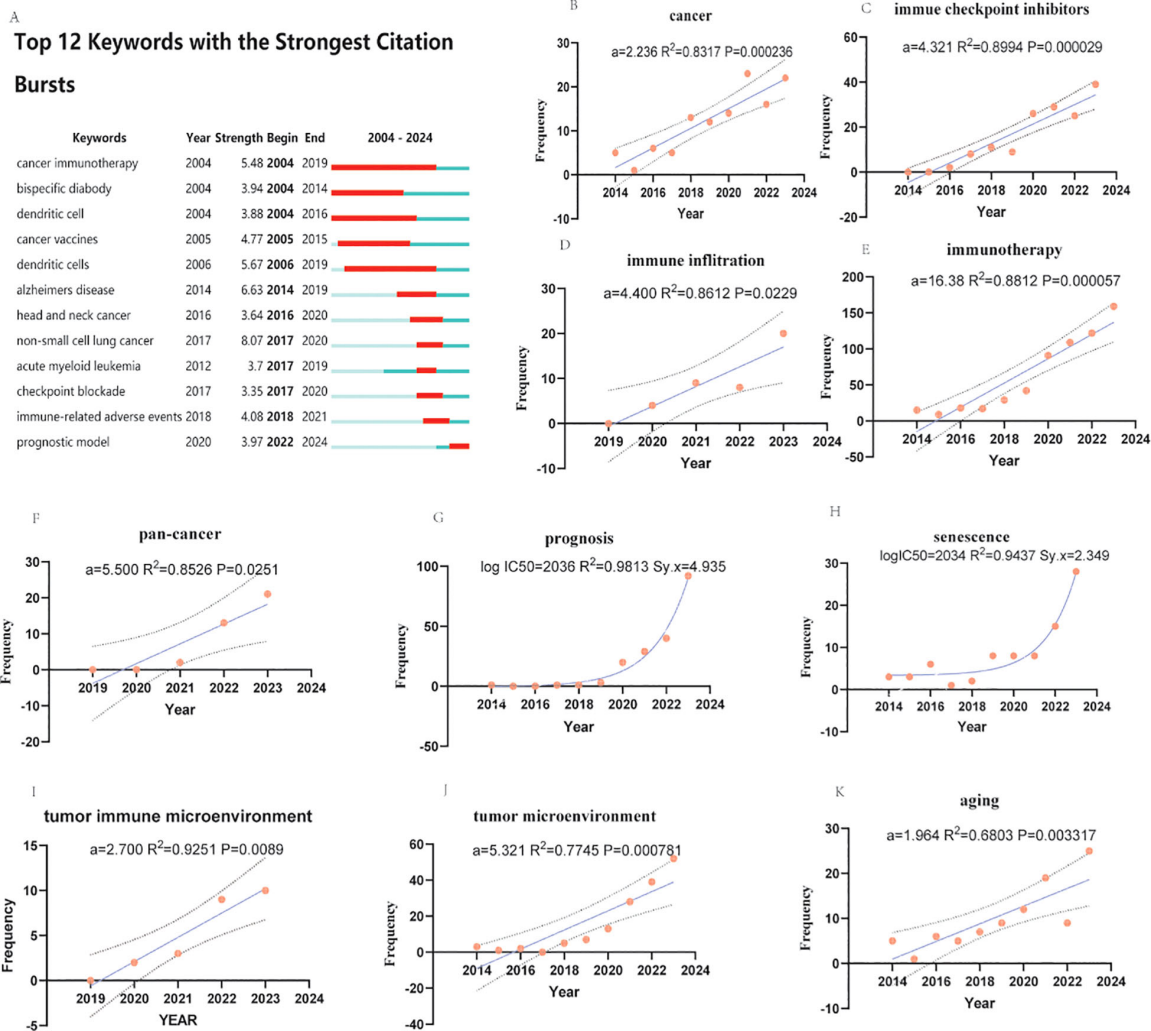
Explosive analysis, evolution, and statistical analysis of research characteristics in aging, cancer, and immunotherapy. **(A)** Explosion and evolution of the top 12 study features with the strongest explosions. The depth in red represents the strength of the explosion. **(B-F)** and **(I-K)** Linear regression analyses of the frequency of annual publications of research features. A represents the slope. r2 denotes the correlation coefficient. p values represent significant differences. **(G, H)** represent nonlinear regression analyses of the frequency of annual issues of research features. (a is regression coefficient. Larger coefficients suggest a stronger effect. Immunotherapy′s much larger coefficient implies it has a far greater impact on the outcome than aging in this study. R² measures how well the predictor explains changes in the outcome. It ranges from 0% to 100%. A higher R² means the predictor is more closely tied to the outcome. These numbers show associations, not proof of cause. Other factors (e.g., lifestyle, genetics) might also play a role but aren′t measured here. logIC50 means the time to reach the middle half of the most frequent author keywords. r2 represents the correlation coefficient. Sy.x represents the standard deviation of the residuals (only the last ten years or five years of data were counted because many terms did not appear in the previous period).

Our analysis revealed that immunotherapy (**Figure 7C**) and prognosis (**Figure 7G**) were the strongest, with aging (**Figure 7K**) as the main representative term in the earlier period, and currently, senescence (**Figure 7H**) is the dominant.

All linear regression analyses (**Figures 7B-F, I-K**) revealed statistically significant differences (p<0.05), especially in terms of search terms, suggesting the soundness of this analytical strategy (**Figures 7B, E**). Moreover, Sy. All nonlinear regression analyses also revealed statistically significant differences (Sy. x<5), and the fit was high and had significant predictive significance (R^2^>0.9).

In linear regression analysis, immunotherapy (**Figure 7C**), the tumor microenvironment (**Figure 7J**) and the pan-cancer model (**Figure 7F**) showed a trend toward significant eruption, and immune inflation (**Figure 7D**) with immune checkpoint inhibitors (**Figure 7C**) showed a more prominent burst trend, whereas the three keywords of aging (**Figure 7K**), the tumor immune microenvironment (**Figure 7I**) and cancer(**Figure 7B**) showed more moderate development, and the latter two terms had high fit (R^2^>0.85), were statistically significant (P<0.01), and were suitable for bottom-up studies.

In the nonlinear regression, both prognosis (**Figure 7G**) and senescence (**Figure 7H**) showed strong trends, which are expected to develop over a long period of time.

### 3.10 Frequency analysis of correlated protein pathway substances and gene studies

**Figure 8:**
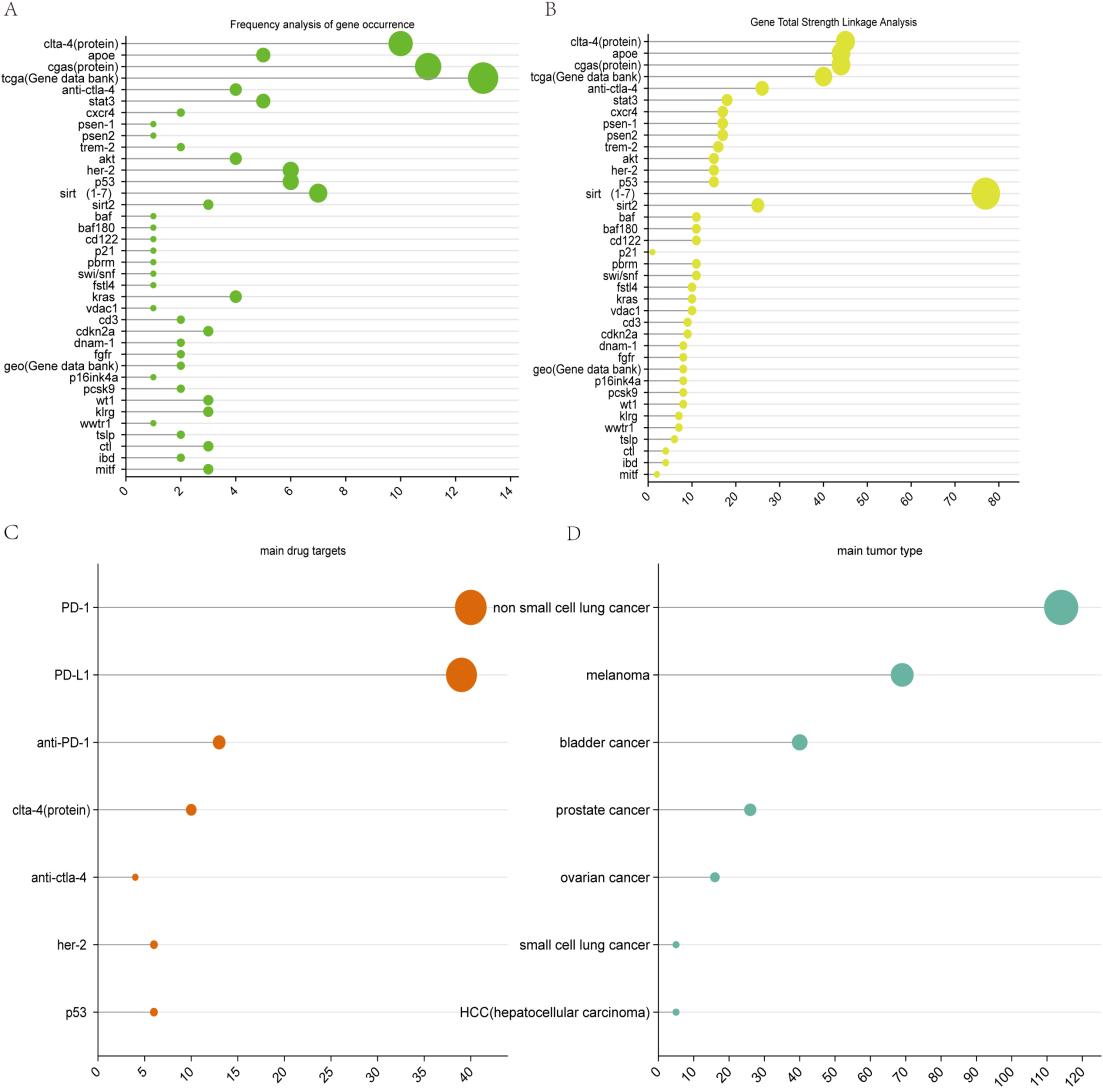
Visualization of genetic analysis maps. **(A)** Frequency analysis of gene occurrence. **(B)** Total strength linkage analysis of genes. **(C)** main drug target. **(D)** main tumor type.

**Figure 8A** shows that, from the frequency of occurrence gene database to the *TCGA* gene database, the gene sequences with the highest number of occurrences and the greatest number of occurrences of *APOE gene* sequences were identified, followed by the *clta-4 genes. sirt* (1–7) *and her-2, p53, akt, kras gene* sequences are well developed. The novel or declining gene sequences are *psen1, psen2, baf, baf180, p21, perm, fstl4*, and other genes, which occur less frequently in this field and have low linkage strengths. As shown in **Figure 8B**, the gene with the strongest linkage to the current research area is the *Sirt1-7 gene* sequence, which is associated mainly with aging, whereas the *clta-4, apoe, cgas, TCGA (gene database), and anti-clta-4 genes* are clusters with strong linkages.

It is expected that the *sirt1-7 gene* and *TCGA databas*e will maintain good development trends in the future.

## 4 Conclusion

The combination of aging therapies with tumor immunotherapies is currently in its preliminary stages. Although senescence has the greatest impact on ICB therapies, mechanistic studies and drug development targeting APOE (to reduce or inhibit APOE expression and thereby reduce immune cell senescence and immunotherapy resistance) and sirt1-7 (to study the common pathway that activates the sirt family of cancer inhibitory pathways and senescence-delaying pathways) may be the key to combining senescence therapies with immunotherapies in the treatment of tumors.

## 5 Discussion

### 5.1 General overview

In recent years, reviews, pathogenesis studies, clinical trials, and other studies on aging, immunotherapy, and elderly patients have been conducted and have received extensive attention from many researchers. This study reveals for the first time the trends of global scientific research published through science and technology on senescence and aging, cancer, and immunotherapy from 2004–2023, including the temporal and spatial distribution of the literature and its contributions; the network linkages of authors, journals, institutions, and countries; and the time of development and overall citations. Keyword clustering, Explosive analysis, and genetic hotspot analysis were also conducted, and corresponding linear and nonlinear analyses were performed by establishing keyword characteristics to predict future research hotspots.

The number of publications and citations in the literature practically reflects the relevant progress and hotness of the field, and the annual fluctuation in the number of publications and citations in the literature was not significant in the past two decades until it significantly increased after 2018. This number has maintained a high growth rate thus far, and the ability to predict the number of publications per year is “y = 22.65x– 45471.16”. The ability to predict the number of citations per year is ‘y = 796.07x–1598985.04’. On the basis of the prediction function, we predict that in 2030, there will be approximately 508 publications in the field as well as 17037 citations. Furthermore, upon in-depth analysis of the landmark literature on the basis of the highest local citation rates, the core of almost all (90%) of this literature points directly to the molecular mechanisms of cellular senescence and tumorigenesis and their potential applications in immunotherapy (e.g., as a therapeutic target, utilization of immune checkpoint inhibitors, and prognostic prediction). The remaining 10% of the literature focuses on population aging and hepatocellular carcinoma, again an area of research closely related to cellular senescence and tumorigenesis. These results suggest that the molecular mechanisms of cellular senescence and tumorigenesis and their application in immunotherapy are important research features in this field. In addition, they were published in high-quality journals in the field, as shown in **Table 1**. The high impact of these studies in related fields further supports that the molecular mechanism of cellular senescence and tumorigenesis and its application in immunotherapy is an important research frontier in this field, confirming the reliability of the above conclusion.

### 5.2 Research hotspots

#### 5.2.1 Immunotherapeutic research

Further visualization of the authors’ keyword hotspot multi-network showed that current research is focused on immunotherapy studies (cluster 3), mainly drug target receptors such as pd-1 (TLS=233) and immune checkpoint inhibitors (TLS=238), and corresponding drugs. Disease models were mainly non-small lung cancer (TLS=260) and melanoma (TLS=178) disease models, and excluded myocarditis (TLS=8) as an unrelated option. This primarily involved immune checkpoint inhibition therapies in tumor immunotherapy.

PD-1 is one of the key coinhibitory receptors expressed on T cells after T-cell activation and tumor cells hijack these inhibitory pathways to evade drug targets by overexpressing PD-L1. Hijacking these inhibitory pathways to evade host immune surveillance, provides a scientific theoretical basis for the clinical application of immune checkpoint inhibitors in oncology (22). Immune checkpoint inhibitors targeting the PD-1/PD-L1 axis, one of the most common immunotherapies, have gained increasing attention for their value in oncology treatment and provide a paradigm to revolutionize the translation of cancer immunotherapy from the laboratory to the clinic (20–24).

Further, in association with cluster 1 (cellular senescence and prognosis), clusters 4 (immunosenescence-related research factors), and 6 (pathophysiological aspects of aging), aging-related research was more closely associated with immune checkpoint inhibition therapy than with over-the-counter cellular immunotherapy and cancer vaccine therapy. This is mainly related to aging-related phenotypes. This is mainly related to the fact that senescence-associated phenotype (SASP) influences the efficacy of immune checkpoint inhibitors (25), and studies have shown that the inhibitory PD-1/PD-L1 immune checkpoint axis appears to play a crucial role in the accumulation of senescent cells, which are protected from PD-1 checkpoint clearance by cytotoxic T cells through overexpression of PD-L1 in the relevant senescent cells, thus promotes the progression of senescence in tissues (26). Teh-Wei Wang et al. showed in the p16-creERT2-tdTomato mouse model as well as related cells by single-cell analysis of p16 cells *in vivo* that PD-L1 expression was associated with higher levels of SASP, and that injection of PD-1 antibody, which reduces the total number of p16 cells *in vivo* and decreases the PD-L1 cell population in a CD^8+^ T-cell-dependent manner, ameliorates a variety of senescence-related phenotypes (27). The above findings suggest that blocking PD-1/PD-L1 checkpoint signals may be a promising anti-aging senolytic therapy. This promises to be a key node for enhancing the immune factors of tumor immunotherapy by de-blocking the protective effects of aging on tumor cells. The development of age-specific strategies to mitigate inhibitory immune checkpoint signals and reduce hyporesponsiveness or exhaustion of aged T cells without compromising antitumor effects represents a significant objective in The development of senescence-specific strategies to mitigate inhibitory immune checkpoint signals and reduce hyporesponsiveness or exhaustion of aged T cells without compromising antitumor effects represents a significant objective in the advancement of these modalities. However, the higher probability of adverse immune-related events that occur in parallel and the impact on patient prognostic models are also a future challenge for this therapy (28).

#### 5.2.2 Cellular senescence

In addition, the results of the temporal distribution of keyword clusters indicate that cellular senescence and prognosis constitute a relatively new research cluster(average year of publication = 2021.4913), which suggests that the progress in this field may be driven by new experimental techniques, data analysis methods (e.g., machine learning), or clinical needs, such as the popularity of whole-genome sequencing and the use of machine learning-based algorithms in cellular apoptosis modeling. The main linkages with cellular senescence are the tumor microenvironment, biomarkers, immune infiltration, and machine learning. Reflects the wide application of multidisciplinary intersections in existing research. The roles of cellular senescence in the tumor microenvironment, associations with biomarkers, immune infiltration, and the construction of predictive models (machine learning) are all central to this research area. Moreover, the trend change from the study of senescence mechanisms to the study of machine learning and prognostic prediction of patients reflects that the current research focus may be gradually shifting from the traditional laboratory exploration of mechanisms to their value in clinical applications. This phenomenon suggests that cellular senescence converges with other hot areas. This convergence represents both the cutting-edge dynamics of the field and reveals potential directions for future research and clinical applications.

On the other hand, owing to the dual role of the tumor suppressor and tumor promoter effects of cellular senescence in cancer development, the study of the effects of cellular senescence on the cancer microenvironment may also serve as a future research focus. *In vitro* culture experiments have shown that senescent cells exhibit a SASP, which transforms senescent fibroblasts into proinflammatory cells with the ability to promote tumor development, which in turn is more likely to cause precancerous lesions and malignant epithelial cells to form tumors (29). Another *in vitro* cell culture experiment on prostate fibroblast senescence and prostate tumors also indicated that aging of the prostate microenvironment leads to increased levels of the paracrine-acting proteins fibroblast growth factor 7, hepatocyte growth factor, and amphiregulin (AREG), which may promote prostate tumor progression (30). In addition, in a study on non-small cell lung cancer (NSCLC), researchers evaluated the expression of lymphocyte immunoglobulin-like receptor B2 (LILRB 2) and its effect on the level of cellular senescence and the behavior of tumor cells, and the results showed that LILRB 2 silencing could significantly increase radiosensitivity in an NSCLC model (31). Other studies on the tumor microenvironment have noted that environmentally mediated resistance is a new form of drug resistance that arises from adaptation between tumor cells and the surrounding microenvironment (32, 33), which also presents new directions and challenges for future research on tumor therapy.

### 5.3 Combining tumor immunotherapy with senescent therapy

Of particular interest are studies and reports on immune infiltration and tumor immune microenvironment, which, although relatively recent (Avg.pub.year:2022), have had considerable research momentum (immune infiltration TLS=98, tumor immune microenvironment TLS=60). Outbreak analysis showed that immune infiltration showed a more prominent outbreak trend. In contrast, the trend of tumor immune microenvironment was more moderate and statistically significant, which is suitable for bottom-up research. As a new hotspot of current research, studies targeting these two keywords are also expected to be integrated with therapies for tumors.

Presently, there are three primary directions in oncological senescence therapies: senolytic therapy, senomorphic therapy, and pro-senescent therapy.

Senolytic therapy involves the use of senolytic to selectively target and eliminate senescent cells. As the most prominent senescence therapy to date, its main mechanism is to promote the apoptosis of senescent cells by targeting key enzymes associated with survival and anti-apoptotic mechanisms, such as p53, p21, Akt (protein kinase B, PKB), and FOXO4 (Forkhead Box O4). Senolytic overcome the inherent resistance of senescent cells to apoptosis by inducing programmed cell death (34). The aim is not a single molecule or biochemical pathway, but rather the broader target of senescent cells. Originating from a 2013 study, such drugs demonstrated effectiveness by employing bafilomycin to promote caspase-dependent lymphoma cell death in a cancer context and cyclophosphamide to induce senescence (35). This discussion highlights the D+Q drug combination, Bcl-2 family inhibitors (ABT-737, ABT-263), and the natural compound fisetin. The initial senolytic identified was a combination of dasatinib (D) and quercetin (Q), both of which are approved for human use. This combination has exhibited the ability to alleviate multiple senescent phenotypes in human adipose progenitor cells or preadipocytes and aged mice (36). Compared to conventional anti-tumor medications, this combination is more specifically a senolytic agent, with most side effects being mitigatable through intermittent dosing (37). Currently, it is under evaluation in several human trials for conditions such as idiopathic pulmonary fibrosis and diabetic nephropathy (38, 39). It should be noted that a recent randomized placebo-controlled trial reported more adverse events (AEs) such as fatigue, nausea, headache, diarrhea, reduced appetite, and malaise in the D+Q group, necessitating further risk assessments in ongoing research (40). Beyond the D+Q combination, ABT-737, an inhibitor targeting Bcl-2 (B-cell lymphoma-2) family members, has also been shown to specifically induce apoptosis in senescent cells within mouse lungs and epidermis by inhibiting BCL-W (B-cell lymphoma-2-like protein 2) and BCL-XL (B-cell lymphoma-extra large) (41). However, issues such as lack of oral bioavailability and low solubility exist. Its derivative, ABT-263, displays superior oral bioavailability and has demonstrated potent tumor-clearing effects in xenograft models of small-cell lung cancer and acute lymphoblastic leukemia (42). It has also been reported that the combination of ABT-263 with radiation therapy has shown better therapeutic efficacy in non-small cell lung cancer (NSCLC) (43). However, ABT-263-induced side effects such as thrombocytopenia, fatigue, and nausea also make this strategy a clinical limitation (44). Finally, the study of natural compounds in inducing senescent cell apoptosis remains a focal point in drug development. Fisetin, a flavonoid found abundantly in fruits and vegetables, has been identified with senolytic activity targeting senescent cells, involving multiple molecular targets and signaling pathways (45). Compared to traditional senolytic drugs, it presents lower hematologic toxicity and more favorable side effects (46). Although its specific efficacy awaits further clinical investigation, fisetin holds promise as a potential targeted agent in senolytic therapies.

Senomorphic therapy primarily aims to inhibit the SASP to block the senescent phenotype without affecting cell death. The mechanisms encompass various biochemical pathways, including mTOR, p38 MAPK, NF-κB, JAK/STAT (Janus kinase-signal transducer and activators of transcription pathway), ROCK (Rho associated coiled-coil forming protein kinase), glucocorticoid receptors, and neutralizing antibodies, which interfere with the pro-inflammatory characteristics of senescent cells and normalize the SASP processes within the senescent microenvironment. Senomorphic drugs, or SASP inhibitors, mainly function by inhibiting pathological SASP secretion rather than eliminating senescent cells (47). Although less popular than apoptotic therapies and requiring long-term usage, anti-senescent therapies may offer a safer, more effective alternative, especially for patients with underlying conditions that make them intolerant to senolytic therapy. Unlike the development of senolytics, the first anti-senescent drugs were serendipitously discovered during experimental studies of other drugs, such as metformin, rapamycin (RAPA), and extracts from rambutan (Nephelium lappaceum) seeds. The earliest report on anti-senescent drugs dates back to 2013, describing a study on metformin’s effects on normal human lung diploid fibroblasts IMR 90 and mouse macrophage cell line RAW264.7 (ATCC). This study revealed that metformin possesses anti-tumor activity, reducing the production of inflammatory cytokines in senescent cells and their response to lipopolysaccharide (LPS), suggesting potential new avenues for cancer prevention or therapy (48). An *in vivo* study based on a disc degeneration model strongly supported this notion (49). The FDA has already initiated research to approve additional indications for metformin (50), which might become a treatment option in anti-cancer therapy. Rapamycin, a novel macrocyclic lactone immunosuppressant often used to prevent organ rejection in kidney transplants, has been reported recently to possess broader biological effects, including anti-senescent and SASP modulation. Experimental reports state that rapamycin and its analogs (e.g., everolimus) extend lifespan in mice (51), inhibit tumor cell growth (52), and reduce the incidence and mortality of tumors (53). *In vitro* studies have shown its capability to lower the secretion of senescence markers and SASPs by senescent cells (54). It is noteworthy, however, that while rapamycin was the first drug to exhibit anti-senescent effects in mammals, the vast majority of research has been conducted in mice or other animal models, and further clinical study is necessary to evaluate its effects on human aging and oncology. The side effects of rapamycin, such as metabolic dysregulation, thrombocytopenia, and impaired wound healing, remain significant concerns (55). Interestingly, certain natural compounds have demonstrated senescence-inhibiting properties, indicating their potential as anti-senescent therapeutics. Rambutan, an edible tropical fruit widely cultivated in Southeast Asia, has been investigated in a 2020 study where its seed extract was shown to reduce SASP mRNA expression levels in human dermal fibroblasts (HDFs), suggesting future applications in treating age-related diseases (56). This hints that exploring drug-related research of natural product extracts may herald new directions in pharmaceutical development.

Finally, pro-senescence therapy, mainly through the induction of the target cells into the senescence state, so that their secretion of SASP factors and thus the use of SASP on the tumor inhibition or enhance the immune system on the clearance of senescent cells. Unlike apoptosis therapy, pro-senescence therapy makes use of immune-mediated clearance of senescent cells, which on the one hand enhances the direct clearance of tumor and premalignant cells by the immune system and on the other hand, also destroys the senescence barrier around tumor cells (57). This therapy involves various pathways such as IL-15 (interleukin -15), CD^4+^ T cells, and PD-1. Despite the relative paucity of research in this direction and the lack of sufficient clinical studies to support it, its excellent inhibitory effect on tumor cells makes it a possible new hotspot for research in the future. It is still the case of metformin, which was found to possess, in addition to its anti-senescent effects, a clear violation of intuition to possess pro-senescent activity (58, 59). Although the mechanism of action is unclear, its acceleration of stress-induced senescence and lowering of the threshold for stress-induced senescence may mimic precancerous stimuli, and thus immune-mediated clearance (‘senescence surveillance’) (58) may be related to its inhibitory effects on cancer. Thus, metformin’s ability to enhance accelerated senescence may be an effective barrier to tumor growth and disease recurrence (60), but more research is needed to explore the mechanisms of this effect.

In addition to this, ciclopirox (CPX) has been found to have apoptosis-inducing or senescence-inducing effects on cervical cancer cells at different glucose levels (61). Since ciclopirox has both mitochondrial oxidative phosphorylation (OXPHOS) inhibitor (62)and iron chelator (63), the researchers investigated its effects on cervical cancer cells at different glucose available concentrations, respectively. The results showed that cyclopiazones had apoptotic effects when glucose availability was limited, whereas cyclopiazones had cell senescence-inducing effects when glucose availability was increased. This interesting finding will probably support the reintroduction of cyclopirox as an optional drug for the treatment of tumors (64).

Most of the above three therapies are mainly focused on inducing senescence and apoptosis in tumor cells or modulating SASP, but if we look beyond the tumor cells themselves to the entire tumor clearance process, a whole new line of research has been born, i.e., the combination of cellular senescence therapies and immunotherapies. All three of these therapies are expected to be combined with tumor immunotherapy through sequential treatment, co-administration, and combination biologics in conjunction with ICB, as well as with passaging cellular therapies.

The PD-1 and PD-L1 axes are important targets for ICB therapies, however, current studies have shown that the inhibitory PD-1/PD-L1 checkpoint pathway works synergistically with immunosuppressive cells; for example, activation of the PD-1 receptor enhances the differentiation of three types of suppressive immune cells, namely, regulatory T-cells, myeloid-derived suppressor cells (MDSCs), and M2 macrophage cells. In the immune microenvironment, immunosuppressive cells are key to preventing immune infiltration, and cytokines secreted by these immunosuppressive cells stimulate the expression of immunosuppressive PD-L1 proteins (26). Thus, blocking PD-1/PD-L1 checkpoint signaling may be a potential anti-senescent senolytic therapy. It also suggests that the development of drugs that block PD-1/PD-L1 checkpoint signaling could be the key to combining ICB therapy with senolytic therapies.

Relay NK cell therapy holds promise as a paradigm for senotherpy therapy by managing senescent cells as well as modulating SASP secretion, in addition to the significant improvement in immune senescence that this approach offers (65). For example, Xiaofeng Tang et al. concluded from a randomized controlled trial of 37 middle-aged men that autologous NK cell administration is an effective approach to significantly alleviate T cell senescence and depletion as well as key components of SASP (66). Paik et al. investigated the effects of sequential NK cell infusion in combination with dopamine-releasing peptide in a mouse model of senescence and showed effective elimination of senescent cells (SNCs) in a variety of tissues and reduction of local and systemic SASP in senescent mice (67).

As for the combination of CAR T-cell therapy with senescence therapy, Corina Amor et al. (16)demonstrated the effective ablation of senescent cells *in vitro* and *in vivo* by identifying the urokinase plasminogen activator receptor (uPAR) as a cell-surface protein that is widely induced during senescence using mice and showed that uPAR-specific CAR T cells were effective in ablating senescent cells *in vitro* and *in vivo*, and the results of the experiment demonstrated that uPAR-targeted CAR T cells prolonged the survival of lung adenocarcinoma mice treated with senescence-inducing drug combinations. Apheresis cell therapy (ACT) using autologous tumor-infiltrating lymphocytes (TIL) is emerging as a curative therapy for advanced cancers (68), but its efficacy may be limited by poor TIL persistence after apheresis (69). This not only implies that CAR T-cells targeting senescent cells could be a therapeutic concept for effective senolytics but also demonstrates the feasibility of combining over-the-counter immune cell therapy (ACT) with senescence therapy.

Furthermore, the induction of SASP secretion to enhance immunotherapy targeting and therapeutic efficacy was shown to be feasible. Pharmacological combinations of MAPK and cell cycle protein-dependent kinase 4/6 (CDK 4/6) inhibitors trigger potent and selective NK cell-mediated responses by inducing SASP secretion of NK cell-recruited chemokines and cytokines that promote NK cell proliferation and activation (70). Moreover, the negative effects induced by chronic SASP (10) can be converted into beneficial results for targeted therapy and may be a potential therapeutic option against certain tumors that are poorly treated with conventional immunotherapy. Numerous preclinical studies have demonstrated the feasibility of combining immunotherapy with senescent therapies and hold the promise of a significant breakthrough from the dilemmas faced by existing immunotherapies, with enhanced efficacy and mitigated side effects based on expanded indications.

Most of the reports on the combination of immunotherapy and senescent therapy focus on preclinical studies, while most of the clinical studies are still in progress, but it is foreseeable that this direction will become one of the hotspots for future research.

It is foreseeable that the combination of senescent therapy and immunotherapy will expand the scope of existing immunotherapies, improve their targeting and efficacy, and reduce tumor recurrence. However, the inherent shortcomings of immunotherapy, such as its susceptibility to multiple factors, uncertain prognosis, and high price, remain to be solved (71). First, the mechanism is complex. The release of multiple factors by tumor cell SASP and the involvement of multiple pathways in the action of these factors make therapeutic regimens targeting a single factor or pathway often seem less effective. Second, there is a lack of uniform and effective markers for senescence or senescence inhibition in tumor cells. The diversity of tumors and the tendency of tumors to mutate themselves have resulted in a lack of uniform markers for different types of tumors, despite the fact that some effective markers of senescence are now available for determining the onset or suppression of senescence. This makes it difficult to judge whether a drug is having an effect when assessing efficacy and regulating drug dosage. Last but not least, there is a lack of relevant preclinical and clinical studies on the combination of these two therapies. Since senescent therapies are still new and most of them are still at the stage of clinical research on non-cancer senescent diseases, there is still a long way to go for the clinical application of combining them with immunotherapy.

### 5.4 Most relevant protein pathway substances and genes

Gene frequency analysis yielded the most frequent protein pathway substance APOE, the Sirtuins family as the protein with the highest linkage strength, and TCGA(The Cancer Genome Atlas) as the most frequent gene database.

APOE is an important component of plasma apolipoproteins and is involved in the conversion and metabolism of lipids and proteins *in vivo*. APOE play an important role in senescent, tumor immunity, and tumor prognosis, and Polymorphisms in the APOE gene have been strongly associated with age-related diseases such as Alzheimer’s disease (72). However, APOE appears to play a negative role in tumor immunotherapy with senescent therapy, which is often used to improve disease outcomes by reducing APOE expression or levels.

Among the mechanisms associated with the promotion of senescence, APOE drives human stem cell senescence by destroying perinuclear heterochromatin through the autophagy-lysosomal pathway (73). For example, tumor-derived APOE induced senescence of TRME and NEU cells, and that the removal of senescent cells neu by HDAC inhibitors could improve the therapeutic efficacy of prostate cancer treatment. Reducing APOE expression or levels to improve disease immunotherapy treatment may hold the key to the future. Monica Xiong et al. found that the anti-human APOE antibody HAE-4 (targeting APOE immunotherapy) reduced cerebral amyloid angiopathy and amyloid plaques while improving cerebral vascular function in animal experiments, which provides experimental evidence for immunotherapy for the treatment of Alzheimer’s disease (74). Chuan Liu (75) et al. explored the negative impact of APOE macrophages on ICI treatment by single-cell RNA sequencing (scRNA-seq) and machine learning methods. They confirmed that the best efficacy of APOE inhibitors in combination with ICI was achieved in a 4T1 nomadic mouse model. Meanwhile, Guangyu Fan et al. (76) found that co-localisation of CD14^+^ APOE^+^ cells and MMP7^+^ (matrix metallopeptidase 7) tumor cells led to increased resistance to immunotherapy in non-small cell lung cancer patients by performing single-cell analysis of clinical data. Interestingly, APOE still seems to have a positive aspect in immunotherapy. Jingjing Wei (77) et al. exploited the property of APOE to effectively cross the blood-brain barrier *in vitro* to reveal APOE-mediated whole-body nano-delivery of granzyme B and CpG for the enhancement of glioma immunotherapy. Peng Xiulan et al. on the other hand suggested that APOE may be a key prognostic molecule with immunomodulatory function in gastric cancer and emphasized the importance of APOE in various cancer types including gastric cancer (78). In summary, the AOPE gene has an important role in cellular senescence, tumorigenesis and development and prognosis of tumor patients, which may point the way to the target of senescence therapy combined with immunotherapy for the co-treatment of tumors.

SIRT (sirtuin) is a NAD-dependent (Nicotinamide Adenine Dinucleotide) histone deacetylase involved in a variety of cellular metabolic processes such as aging, proliferation, apoptosis, and DNA repair. Seven members of this enzyme family have been suggested as potential targets for the treatment of human conditions, including cancer and aging (79). Recently, Bi, Shijia et al. elucidated the co-target genes of the seven members of the sirtuins family, and the placenta-specific protein PAPPA (Pregnancy-Associated Plasma Protein A) can be ectopically expressed in response to the deletion of any of the sirtuin proteins, which leads to cellular senescence (80).

SIRT1 is the most studied NAD-dependent histone deacetylase, and the SIRT1 signaling pathway is closely related to many important pathways that regulate senescence, and also plays an important role in cancer development (81).

SIRT1 is directly or indirectly involved in the regulation of the senescence signaling pathway through the deacetylation of certain key proteins (currently mainly six proteins corresponding to six different pathways, NF-KB (nuclear factor kappa-B), AMPK (AMP-activated protein kinase), mTOR, p53, PGC1α (peroxisome proliferator-activated receptor gamma co-activator-1 alpha), and FoxOs (Forkhead box O)) (82), which can delay or even counteract cellular senescence. This lays the foundation for SIRT1 as an anti-aging therapy. For example, NF-κB is a major transcription factor in cells, which is associated with inflammation and senescence-related diseases, and corresponding research evidence suggests that sustained activation of NF-κB promotes cellular senescence (83), while SIRT1 can negatively regulate the action of NF-κB, thereby counteracting senescence (84). Cuichen et al. found that in the annual fish Nothobranchius guentheri that resveratrol detected in the annual fish Nothobranchius guentheri could extend lifespan by reducing senescence-related secretory phenotypes through the SIRT1/NF-κB pathway in the gut (85). Although the SIRT1-NF-κB pathway has significant anti-senescence effects, its mechanisms associated with cancer cell senescence and immune cell senescence remain to be further explored. Chen Peng et al. found that the activation of miR-34a-mediated SIRT1/mTOR signaling pathway was attenuated by urinary phospholipid A. Recent studies have shown that mTOR can regulate the senescence process by acting on autophagy and S6K (S6 kinase) downstream of TORC1 (TOR complex 1), and that the TORC1-S6K-Syx13 signaling pathway plays a central role in inflammatory senescence, immune senescence, and lifespan regulation (86). This result suggests that SIRT1 and mTOR are expected to counteract immune senescence and improve the efficacy of tumor immunotherapy. In addition, Sirtuin1 inhibits aging and aging-related diseases by driving metabolic adaptations in the calorie restriction pathway (87).

However, during carcinogenesis, SIRT 1 has both pro- and oncogenic effects, which provides a theoretical basis for SIRT1 as a target for tumor immunotherapy in combination with anti-ageing therapies. One of them, the SIRT1-P53 axis, is thought to play a central role in tumorigenesis and development, and may also be an important pathway for future use as a potential tumor immunotherapy in combination with ageing therapies. Remco S Derr et al. found that the expression level of SIRT1 was significantly elevated in breast cancer (88). And one of the important signaling pathway axes, i.e. SIRT1-P53 axis. This is due to the fact that SIRT1 can deacetylate p53 during tumorigenesis and development, but it makes SIRT1 a potential oncogene and inhibits the anti-tumor effect of p53 (89). In addition, Leng Shuai et al. found that SIRT1 synergistically regulates pancreatic cancer stem cell properties and promotes tumorigenesis with the CRL4B (Cullin 4B (CUL4B)-Ring E3 ligase) (87), in which SIRT1/CUL4B promotes a variety of CSC (cancer stem cell) properties, including increased expression of stemness marker expression and sphere formation, and could also be a potential target for tumor immunotherapy. At the same time, SIRT1 exerts opposite anti-cancer effects in glioblastoma of the nervous system. Studies have shown that inhibition of Sirtuin overexpression suppresses cell growth and induces apoptosis in glioma cell lines U87 and T98G (T98G-shNRF2) (90), whereas X. Chen et al. demonstrated that SIRT1 activated by AROS induces a depletion-dependent activation of ATF3 plus NAD+, which sensitizes glioma cells to iron death (91). In contrast, NF-κB-mediated signaling is the main modulator of the pro-inflammatory effects of SASP in senolytics targeting SASP. In contrast, SIRT1 regulation of NF-KB may favor the progression of this therapy and simultaneously regulate the corresponding cellular senescence, enhancing the efficacy of immunotherapy. Current mechanistic studies have found that SIRT1, SIRT3 and SIRT6 are protective against vascular senescence, while the anti-aging effects of other SIRTs are still under investigation (92).

Meanwhile, related studies have shown that SIRT3, SIRT4 and SIRT6 also inhibit the Warburg effect and exert anticancer effects by regulating components of the phosphatidylinositol-3 kinase pathway, which is central to cancer metabolism (93).

In conclusion, the sirtuins family plays a key role in the development of aging and cancer, however, the complexity of the multiple mechanisms through which they regulate the corresponding diseases in different cells may be an obstacle to the progress of the current research. Nevertheless, elucidating and applying the relevant regulatory mechanisms of the sirtuins family in both cancer and aging pathologies is expected to pave the way for the combination of immunotherapy for tumors and therapies for aging. However, the elucidation and application of sirtuins in both cancer and aging is expected to pave the way for combining tumor immunotherapy and aging therapy.

### 5.5 Limitations

In this study, only the WoSCC database was utilized, and other databases such as PubMed, Scopus, and CNKI were excluded to avoid weighted repetition and maintain clarity. However, this approach may have resulted in the omission of key studies indexed exclusively in those platforms, introducing selection bias and limiting the comprehensiveness of the literature review. Moreover, tools like CiteSpace and VOSviewer, while powerful for bibliometric analysis, rely on unique keyword frequency screening mechanisms that prioritize high-frequency terms, potentially overlooking emerging keywords or niche topics that have not yet gained substantial citation counts. This screening process may bias the analysis toward well-established research areas at the expense of burgeoning fields, and the clustering based on author-supplied keywords further compounds this issue due to the lack of standardization and possible overlap or omission of terms. Additionally, bibliometric analysis cannot evaluate the quality of individual studies, as it primarily relies on citation counts, which may not accurately reflect scientific rigor or relevance (94). This is particularly problematic for recent high-quality papers with limited citations, as their impact may be underrepresented. Temporal citation burst analysis is another limitation, as trends are influenced by the selected 20-year timeframe, with citation burst periods potentially skewing the significance of certain topics. Nonlinear regression, despite its flexibility in modeling complex relationships, has its own drawbacks. Unlike linear regression, it does not produce p-values, and while Sy.x (the standard deviation of residuals) was used as a credibility measure, it may not fully account for uncertainties in predictive accuracy. Nonlinear regression models can also be highly sensitive to assumptions and specifications, which may impact the generalizability of predictions. Furthermore, some data in the citation burst analysis existed only in one of the 20-year periods, which may have created prediction biases due to the existence of stable periods and citation growth bursts within the selected timeframe. In addition, bibliometric analyses often lack the contextual depth necessary to explore nuanced discussions of molecular mechanisms or experimental details, as space and scope constraints exclude qualitative content. More often than not, valuable content may have been excluded due to such limitations, hindering a comprehensive understanding of the analyzed topics. Finally, the reliance on visualization tools can introduce challenges, such as overlapping or irrelevant terms in keyword clusters, which may obscure the clarity of network linkages. These limitations cumulatively affect the robustness and generalizability of the findings, as database exclusion and keyword biases may overlook emerging trends, while the inability to assess study quality could overemphasize less rigorous but highly cited studies. Prediction biases from citation burst analysis, and uncertainties in nonlinear modeling, further necessitate cautious interpretation of research trends and highlights. Therefore, while this study provides an essential bibliometric overview, it is crucial to supplement these findings with in-depth systematic reviews and experimental studies for validation and further context.

## Data Availability

The raw data supporting the conclusions of this article will be made available by the authors, without undue reservation.
